# Signal Peptide Features Determining the Substrate Specificities of Targeting and Translocation Components in Human ER Protein Import

**DOI:** 10.3389/fphys.2022.833540

**Published:** 2022-07-11

**Authors:** Sven Lang, Duy Nguyen, Pratiti Bhadra, Martin Jung, Volkhard Helms, Richard Zimmermann

**Affiliations:** ^1^ Medical Biochemistry and Molecular Biology, Saarland University, Homburg, Germany; ^2^ Center for Bioinformatics, Saarland University, Saarbrücken, Germany

**Keywords:** endoplasmic reticulum, protein targeting, protein translocation, signal peptides, Sec61 complex, human cells, siRNA-mediated depletion of single targeting or transport components, label-free quantitative proteomics in combination with differential protein abundance anlysis

## Abstract

In human cells, approximately 30% of all polypeptides enter the secretory pathway at the level of the endoplasmic reticulum (ER). This process involves cleavable amino-terminal signal peptides (SPs) or more or less amino-terminal transmembrane helices (TMHs), which serve as targeting determinants, at the level of the precursor polypeptides and a multitude of cytosolic and ER proteins, which facilitate their ER import. Alone or in combination SPs and TMHs guarantee the initial ER targeting as well as the subsequent membrane integration or translocation. Cytosolic SRP and SR, its receptor in the ER membrane, mediate cotranslational targeting of most nascent precursor polypeptide chains to the polypeptide-conducting Sec61 complex in the ER membrane. Alternatively, fully-synthesized precursor polypeptides and certain nascent precursor polypeptides are targeted to the ER membrane by either the PEX-, SND-, or TRC-pathway. Although these targeting pathways may have overlapping functions, the question arises how relevant this is under cellular conditions and which features of SPs and precursor polypeptides determine preference for a certain pathway. Irrespective of their targeting pathway(s), most precursor polypeptides are integrated into or translocated across the ER membrane via the Sec61 channel. For some precursor polypeptides specific Sec61 interaction partners have to support the gating of the channel to the open state, again raising the question why and when this is the case. Recent progress shed light on the client spectrum and specificities of some auxiliary components, including Sec62/Sec63, TRAM1 protein, and TRAP. To address the question which precursors use a certain pathway or component in intact human cells, i.e., under conditions of fast translation rates and molecular crowding, in the presence of competing precursors, different targeting organelles, and relevant stoichiometries of the involved components, siRNA-mediated depletion of single targeting or transport components in HeLa cells was combined with label-free quantitative proteomics and differential protein abundance analysis. Here, we present a summary of the experimental approach as well as the resulting differential protein abundance analyses and discuss their mechanistic implications in light of the available structural data.

## 1 Introduction

### 1.1 Protein Biogenesis at the Endoplasmic Reticulum

**FIGURE 1:**
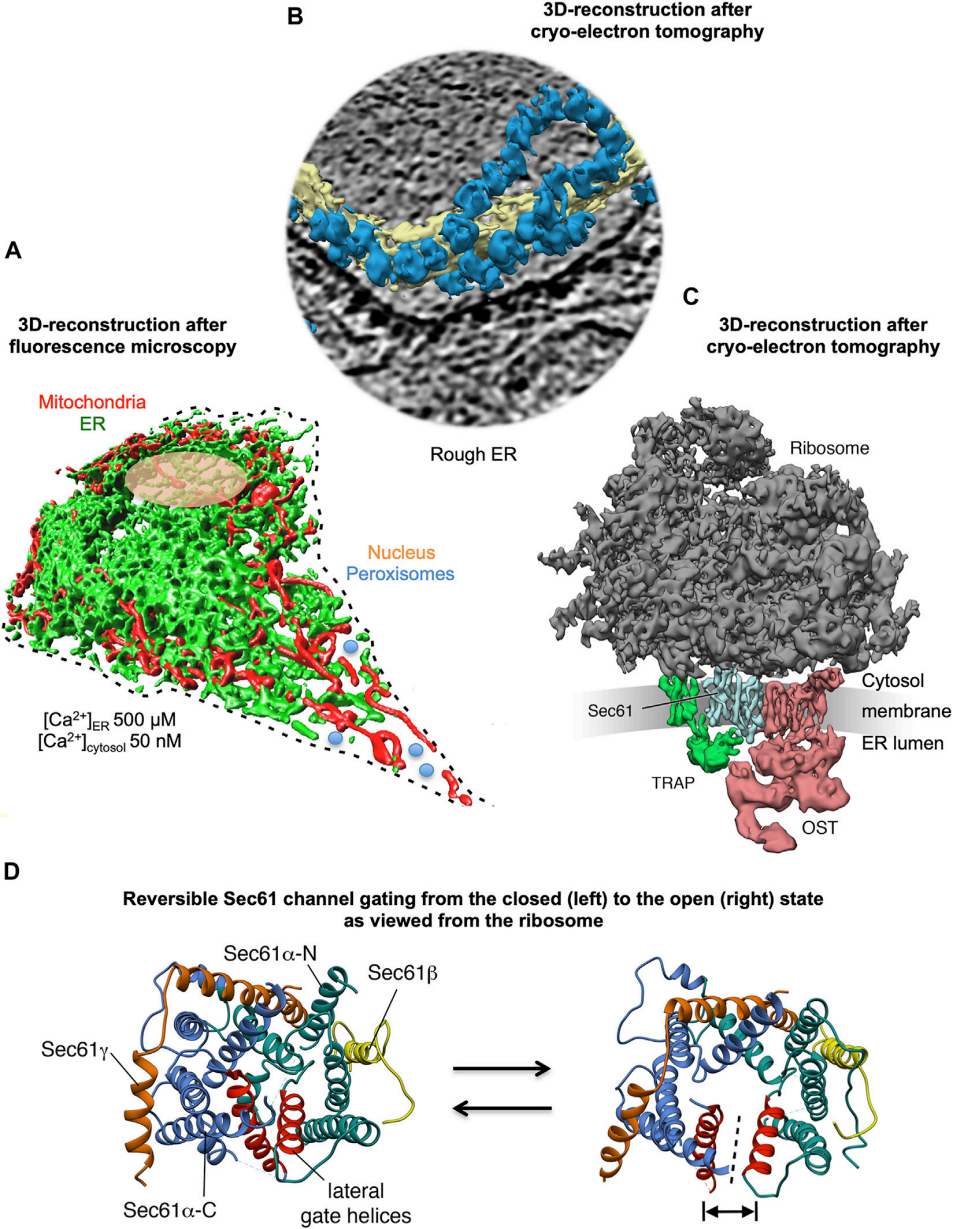
3D reconstructions of a nucleated mammalian cell, a section of rough ER, and a ribosome-bound Sec61 translocon. **(A)** 3D reconstruction after live cell fluorescence imaging, following import of GFP into the ER and of RFP into the mitochondria. The reconstruction was artificially complemented by a dashed line for the plasma membrane, by an orange ellipse for the nucleus, and by a couple of blue circles for peroxisomes. Typical concentrations of free Ca^2+^ are given for cytosol and ER in a resting cell. **(B)** 3D reconstruction of cellular rough ER after CET of a slice through the respective tomogram. ER membranes are shown in yellow, 80S ribosomes in blue. **(C)** 3D reconstruction of the native ribosome-translocon complex in rough microsomes. The membrane density was removed for better visibility of membrane integral parts of the translocon complex. TMHs in Sec61 complex, TRAP and OST can be distinguished. Helix 51 of an rRNA ES and ribosomal protein eL38 represent contacts of TRAPγ, but are hidden by other ribosomal densities. **(D)** The concept of reversible gating of the heterotrimeric Sec61 complex by SPs and allosteric effectors. The Sec61 channel is shown in its modeled closed (left) and open (right) conformational states, as viewed from the cytosol. These two states are suggested to be in a dynamic equilibrium with each other. The fully open state of the Sec61 channel allows the initial entry of precursor polypeptides from the cytosol into the ER lumen and ER membrane, respectively, and is experimentally observed as cleavage of SPs by signal peptidase on the lumenal side of the ER membrane. In addition, it allows the passive efflux of Ca^2+^ from the ER lumen into the cytosol and can be observed in live cell Ca^2+^ imaging in cytosol and ER lumen (Erdmann et al., 2011; Schäuble et al., 2012). Ca^2+^ efflux may also be possible in the expected transition state (not shown), which may be identical to the so-called primed state and is induced by ribosomes in cotranslational- and by Sec62/Sec63 in posttranslational-transport. The Figure and Figure legend were adapted from Sicking et al. (2021a).

In analogy to the division of the human body into various organs, the nucleated human cell is divided into different compartments, the cell organelles. Organelles are surrounded and, thus, separated from the cytosol by phospholipid bilayers (Figure 1A). The vast majority of the roughly 30,000 types of polypeptides of human cells is synthesized in the cytosol. Therefore, the proteins of the various organelles have to be targeted to the specific organelles and, subsequently, inserted into or translocated across the organelle membrane(s). Protein import into the organelle network termed endoplasmic reticulum (ER) is the first step in the biogenesis of about one third of the different soluble and membrane proteins of human cells (Gemmer and Förster, 2020; O'Keefe et al., 2021a; Pool, 2022; Tirincsi et al., 2022a). The hallmarks of this process were first established about 70 years ago by Palade et al., who also described different ER morphologies, or -as these are termed today- domains (Palade and Porter, 1954; Palade, 1975). From their electron microscopic images these authors concluded that the ER represents a “continuous, tridimensional reticulum” consisting of “cisternae,” which appear to communicate freely with the “tubules” (Palade and Porter, 1954; Palade, 1975). Furthermore, Palade et al. wrote that “although such cisternae may assume considerable breadth they seem to retain, in general, a depth of ∼50 µm” and “the surface of the latter appears to be dotted with small, dense granules that cover them in part or in entirety” (Palade and Porter, 1954; Palade, 1975). Today, those original domains of the ER are referred to as rough sheets and smooth tubules, where rough and smooth refers to the presence or absence of the dense granules observed by Palade et al. (Palade and Porter, 1954; Palade, 1975), i.e., ribosomes or polysomes which are attached to the cytosolic ER surface (Figure 1B) (Shibata et al., 2006; Shibata et al., 2010; Westrate et al., 2015; Nixon-Abell at el., 2016; Valm et al., 2017).

Originally, the roughly 10,000 soluble and membrane proteins that first enter the ER in the course of their biogenesis were known to fulfill their functions in the membrane or lumen of the ER plus the nuclear envelope, or in one of the organelles of the pathways of endo- and exocytosis (i.e., ERGIC, Golgi apparatus, endosome, lysosome, trafficking vesicles), or at the cell surface as secretory- or plasma membrane-proteins. With the exception of resident proteins of the ER, most of the correctly folded and assembled proteins are transported to their functional location by trafficking vesicles, which bud from sub-domains of the tubular ER that are termed exit sites (ERES) (Raote et al., 2018; Raote et al., 2020)*.* In recent years, however, an increasing number of membrane proteins destined to lipid droplets, peroxisomes or mitochondria was observed to be first targeted to and inserted into the ER membrane prior to their integration into budding lipid droplets or peroxisomes or prior to their delivery to mitochondria via the ER-SURF pathway (Hansen et al., 2018; Schrul and Schliebs, 2018; Jansen and Klei, 2019; Dhimann et al., 2020; Goodman, 2020; Koch et al., 2021; Lalier et al., 2021). Interestingly, the budding of lipid droplets and peroxisomes also occurs in sub-domains of the tubular ER, which may be spatially or physically related to ERES (see below) (Schrul and Kopito, 2016; Song et al., 2021; Zimmermann et al., 2021). Moreover, several cytosolic proteins are synthesized on ER-bound ribosomes (Seiser and Nicchitta, 2000; Potter et al., 2001; Pyhtila et al., 2008; Reid and Nicchitta, 2012; Calvin et al., 2014; Berkovits and Mayr, 2015; Ma and Mayr, 2018).

### 1.2 Endoplasmic Reticulum Targeting Mechanisms

**FIGURE 2:**
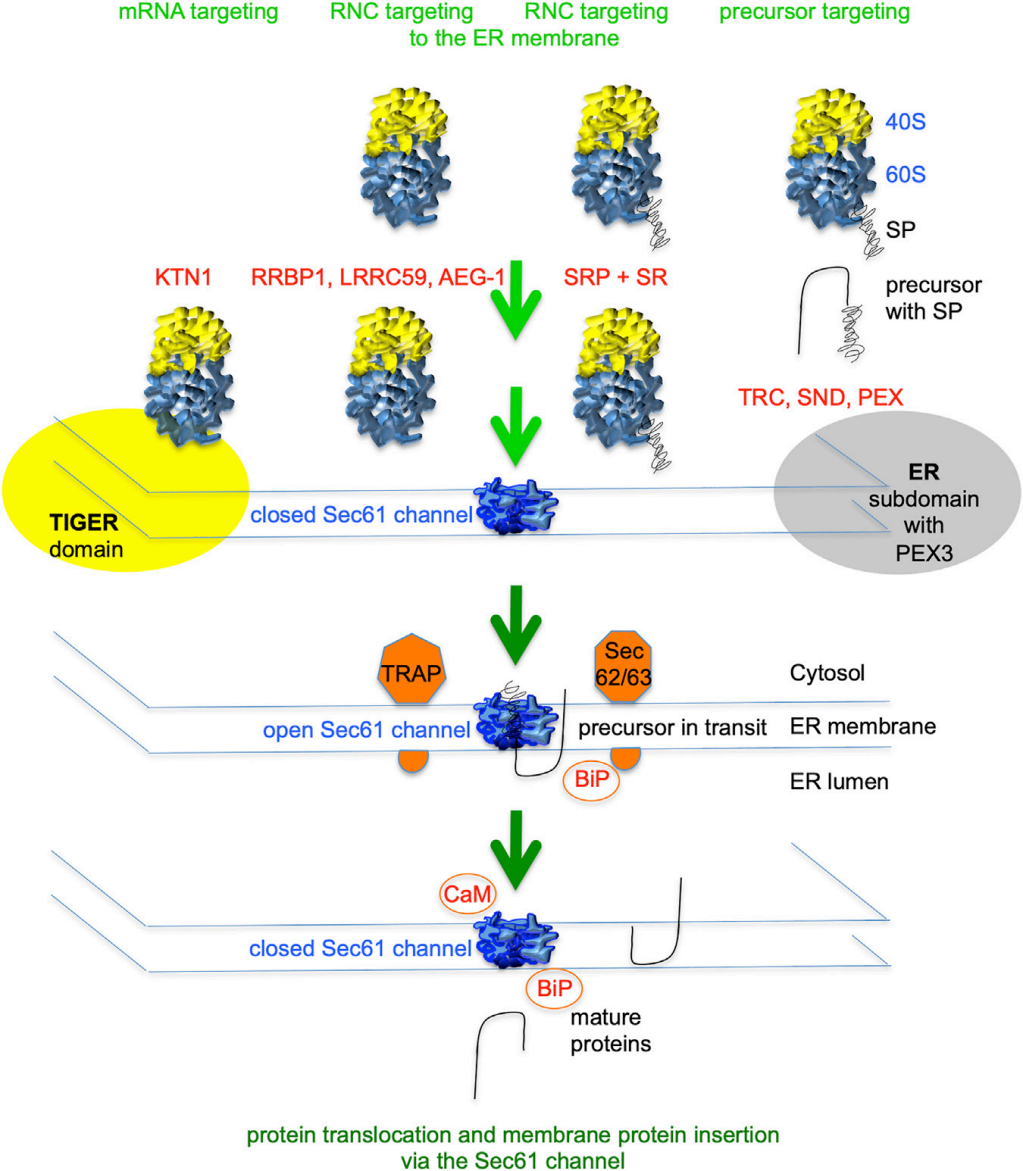
Flow diagram for signal peptide-dependent import of precursor polypeptides into the human endoplasmic reticulum (ER). ER import of most precursor polypeptides involves the Sec61 channel in the ER membrane, which mediates membrane insertion of membrane proteins and translocation of soluble proteins with N-terminal signal peptides (SPs). Typically, SPs of nascent precursor polypeptides are cotranslationally targeted to the Sec61 complex in the ER membrane by SRP and its dimeric receptor in the ER membrane (SR). Others are targeted co- or posttranslationally by the TRC-, PEX19/PEX3- or hSnd2/hSnd3-pathway. Furthermore, there are components for mRNA- and/or RNC-targeting located in the ER membrane. Additional ER membrane proteins support Sec61 channel gating to the open state (TRAP or Sec62/Sec63) or membrane protein insertion, such as EMC (not shown). Channel gating to the closed state can be supported by cytosolic Ca^2+^-CaM or BiP in the ER lumen (Erdmann et al., 2011; Schäuble et al., 2012). The green arrows symbolize progress of the import reaction. See Table 1 for a complete list of proteins that are involved in ER protein import.

Typically, protein import into the ER involves ER membrane targeting as the first step and insertion of nascent or fully-synthesized membrane proteins into or translocation of soluble precursor polypeptides across the ER membrane as the second step (Figures 1C,D). These two processes depend on cleavable amino-terminal signal peptides (SPs) or non-cleavable and more or less amino-terminal transmembrane helices (TMHs), which, by definition, both serve as import determinants in the precursor polypeptides (von Heijne, 1985; von Heijne, 1986; von Heijne and Gavel, 1988; Goder and Spiess, 2003; Goder et al., 2004; Hegde and Bernstein, 2006; Baker et al., 2017; Armenteros et al., 2019). In general, the Sec61 complex in the ER membrane represents the entry point for most of these precursor polypeptides into the organelle (Figures 1, 2; Table 1) (Görlich et al., 1992b; Görlich and Rapoport, 1993; Hartmann et al., 1994; Simon and Blobel, 1991; Beckmann et al., 2001; Wirth et al., 2003; van den Berg et al., 2004; Pfeffer et al., 2012; Pfeffer et al., 2014; Pfeffer et al., 2015; Voorhees et al., 2014, Voorhees and Hegde, 2016). However, membrane insertion of some precursors of membrane proteins can be facilitated by alternative membrane protein insertases and components such as the ER membrane protein complex (EMC), TMCO1 complex, and WRB/CAML (Shurtleff et al., 2018; Chitwood et al., 2018; Pleiner et al., 2020; Bai et al., 2020; O'Donnell et al., 2020; Wang et al., 2016; Anghel et al., 2017; McGilvray et al., 2020). Notably, the latter has its main role in the membrane insertion of tail anchored (TA) membrane proteins (Borgese and Fasana, 2011; Yamamoto and Sakisaka, 2012; Borgese et al., 2019). Together with its cytosolic interaction partners, the latter can also facilitate targeting of precursor polypeptides to the Sec61 complex, as apparently do the SRP/SR-, PEX19/PEX3-, and SND-targeting pathways (Figure 2) (Meyer and Dobberstein, 1980a; Meyer and Dobberstein, 1980b; Gilmore et al., 1982a; Gilmore et al., 1982b; Tajima et al., 1986; Siegel and Walter, 1988; Ng et al., 1996; Egea et al., 2005; Gamerdinger et al., 2015; Aviram et al., 2016; Casson et al., 2017; Haßdenteufel et al., 2017; Haßdenteufel et al., 2018; Haßdenteufel et al., 2019; Hsieh et al., 2020; Jomaa et al., 2021). Targeting of certain peroxisomal membrane proteins and some but not all hairpin membrane proteins of lipid droplets or the ER and their insertion into the ER membrane was found to involve cytosolic PEX19 and PEX3 in the ER membrane (Schrul and Kopito, 2016; Yamamoto and Sakisaka, 2018; Leznicki et al., 2021).

**TABLE 1:** Protein transport components/*complexes,* associated proteins in HeLa cells and linked diseases.

Component/subunit	Abundance^a^	Location^b^	Linked Diseases
for ER targeting			
#LRRC59 (LRC59, p34)^c^	2480	ERM	
#RRBP1 (p180)	135	ERM	Hepatocellular Carcinoma, Colorectal Cancer
KTN1 (Kinectin 1)	263	ERM	
AEG-1 (LYRIC, MTDH)	575	ERM	
#*NAC* ^d^	1412	C	
- NAC α			
- NAC β			
*#SRP*		C	
- SRP72	355		Aplasia, Myelodysplasia
- SRP68	197		
- SRP54	228		Neutropenia, Pancreas Insufficiency
- SRP19	33		
- SRP14	4295		
- SRP9	3436		
- 7SL RNA			
*SRP receptor*		ERM	
- SRα (docking protein)	249		
- SRβ	173		
Calmodulin	9428	C	
hSnd1	unknown	ERM	
*Snd receptor*			
- hSnd2 (TMEM208)	81		
- §hSnd3 (TMEM109)	49		
PEX19	80	C	Zellweger Syndrome
PEX3	103	ERM,PexM	Zellweger Syndrome
PEX16	9	ERM,PexM	Zellweger Syndrome
for ER targeting plus membrane integration			
*#Bag6 complex*		C	
- TRC35 (Get4)	171		CDG
- Ubl4A	177		
- Bag6 (Bat3)	133		Lung cancer
SGTA	549	C	Breast cancer, Lung cancer
TRC40 (Asna1, Get3)	381	C	CDG
*TA receptor*		ERM	
- CAML (CAMLG, Get2)	5		CDG
- WRB (CHD5, Get1)	4		Congenital Heart Disease
for ER membrane integration			
*ERM protein complex*		ERM	
- EMC1	124		Visual disorders
- EMC2	300		
- EMC3	270		
- EMC4	70		
- EMC5 (MMGT1)	35		
- EMC6 (TMEM93)	5		
- EMC7	247		
- EMC8	209		
- EMC9	1		
- EMC10	3		Developmental delay
#§*TMCO1 complex*		ERM	Glaucoma, Cerebrofaciothoracic Dysplasia
- TMCO1	2013		
- Nicalin	99		
- TMEM147	21		
- CCDC47 (Calumin)	193		
- NOMO	267		
*PAT complex*	193	ERM	
- PAT10 (Asterix)			
- CCDC47 (Calumin)			
for ER membrane integration plus translocation			
*#§Sec61 complex*		ERM	
- Sec61α1	139		Diabetes^e^, CVID^f^, TKD, Neutropenia
- Sec61β	456		PLD, Colorectal cancer
- Sec61γ	400		GBM, Hepatocellular carcinoma
#Sec62/Sec63		ERM	Breast-, Prostate-, Cervix-, Lung-Cancer
- Sec62 (TLOC1)	26		
- Sec63 (ERj2)	168		PLD, Colorectal cancer
#ERj1 (DNAJC1)	8	ERM	
#TRAM1	26	ERM	
TRAM2	40	ERM	
*#TRAP*		ERM	
- TRAPα ((SSR1)	568		
- TRAPβ (SSR2)			
- TRAPγ (SSR3)	1701		CDG, Hepatocellular Carcinoma
- TRAPδ (SSR4)	3212		CDG
#RAMP4 (SERP1)		ERM	
for folding plus assembly			
*ER Chaperones*			
- BiP (Grp78, HSPA5)	8253	ERL	HUS
- Calreticulin (CaBP3, ERp60)	14521	ERL	
- #Calnexin_palmitoylated_	7278	ERM	
- ERj3 (DNAJB11)	1001	ERL	PKD
- ERj4 (DNAJB9)	12	ERL	
- ERj5 (DNAJC10)	43	ERL	
- ERj6 (DNAJC3, p58^IPK^)	237	ERL	Diabetes, Neurodegeneration
- ERj7 (DNAJC25)	10	ERM	
- ERj8 (DNAJC16)	24	ERM	
- ERj9 (DNAJC22)		ERM	
- Grp94 (CaBP4, Hsp90B1)	4141	ERL	
- Grp170 (HYOU1)	923	ERL	Hyperinsulinismus, Allergic Asthma
- Sil1 (BAP)	149	ERL	MSS
for covalent modification			
*#Oligosaccharyltransferase (OST-A)*		ERM	
- RibophorinI (Rpn1)	1956		
- RibophorinII (Rpn2)	527		
- OST48	273		CDG
- Dad1	464		
- OST4			
- TMEM258			
- Stt3A*	430		CDG
- DC2			
- Kcp2			
*Oligosaccharyltransferase (OST-B)*		ERM	
- RibophorinI (Rpn1)	1956		
- RibophorinII (Rpn2)	527		
- OST48	273		
- Dad1	464		CDG
- OST4			
- TMEM258			
- Stt3B*	150		CDG
- TUSC3			CDG
- MagT1	33		
*Signal peptidase (SPC-A)*		ERM	
- SPC12	2733		
- SPC18* (SEC11A)			
- SPC22/23	334		
- SPC25	94	ERM	
*Signal peptidase (SPC-C)*			
- SPC12	2733		
- SPC21* (SEC11C)			
- SPC22/23	334		
- SPC25	94		
*GPI transamidase (GPI-T)*		ERM	
- GPAA1	9		
- PIG-K	38		
- PIG-S	86		
- PIG-T	20		
- PIG-U	42		

a Here, abundance refers to the concentration (nM) of the respective protein in HeLa cells, as reported by Hein et al. (2015).

b Localization refers to the functional intracellular localization(s) of the respective protein, i.e., C, Cytosol; ERL, ER lumen; ERM, ER membrane; PexM, Peroxisomal membrane.

c Alternative protein names are given in parentheses.

d Complexes are indicated by italics. Abbreviations for protein names: EMC, ER membrane (protein) complex; GET, Guided entry of tail-anchored proteins; GPI, Glycosylphosphatidylinositol; NAC, Nascent polypeptide-associated complex; OST, Oligosaccharyltransferase; SEC, (Protein involved in) secretion; SND, SRP-independent; SPC, signal peptidase; SR, SRP receptor; SRP, signal recognition particle; SSR, signal sequence receptor; TA, tail anchor; TMEM, Transmembrane (protein); TRAM, translocating chain-associating membrane (protein); TRAP, Translocon-associated protein; TRC, transmembrane recognition complex.

e Diabetes was linked to the particular protein in mouse.

f Abbreviation for diseases, i.e., CDG, Congenital disorder of glycosylation; CVID, Common variable immunodeficiency; GBM, Glioblastoma multiforme; HUS, Hemolytic-uremic syndrome; MSS, Marinesco-Sjögren syndrome; PKD, Polycystic kidney disease; PLD, Polycystic liver disease; TKD, tubulointerstitial kidney disease, as reported by Sicking et al. (2021a).

*Indicates enzymatic activity.

# indicates ribosome association.

§Indicates ion channel activity.

We note that Calnexin, ERj1, Sec61ß, Sec63, SRα, TRAM1, and TRAPα were shown to be subject to phosphorylation.

In addition, there is targeting of mRNAs to the Sec61 complex that depends on receptors for mRNAs (such as KTN1) (Ong et al., 2003; Ong et al., 2006), or receptors for ribosome nascent chain complexes, where the nascent polypeptide chains are not yet long enough to interact with SRP (such as RRBP1, LRRC59, and AEG-1) (Figure 2) (Savitz and Meyer, 1990; Tazawa et al., 1991; Ohsumi et al., 1993; Savitz and Meyer, 1993; Calvin et al., 2014; Hsu et al., 2018). In contrast to SRP/SR, these mRNA targeting mechanisms are nucleic acid based and may deliver essentially every kind of mRNA to the ER surface, including mRNAs coding for soluble proteins of the cytosol or the mitochondrial and peroxisomal matrix (Seiser and Nicchitta, 2000; Potter et al., 2001; Calvin et al., 2014; Berkovits and Mayr, 2015; Ma and Mayr, 2018). In the case that the mRNA codes for a cytosolic or matrix protein, the heterodimeric cytosolic protein nascent polypeptide-associated complex (NAC) can get access to the amino-terminus of the nascent polypeptide and trigger its release from Sec61 and the simultaneous release of the ribosome from the Sec61 complex (Wiedmann et al., 1994; Moeller et al., 1998; Gamerdinger et al., 2019; Hsieh et al., 2020). If the mRNA codes for a precursor polypeptide with SP or TMH, however, the latter may spontaneously interact with the Sec61 channel or the productive interaction may be facilitated by one of the protein targeting components.

**FIGURE 3:**
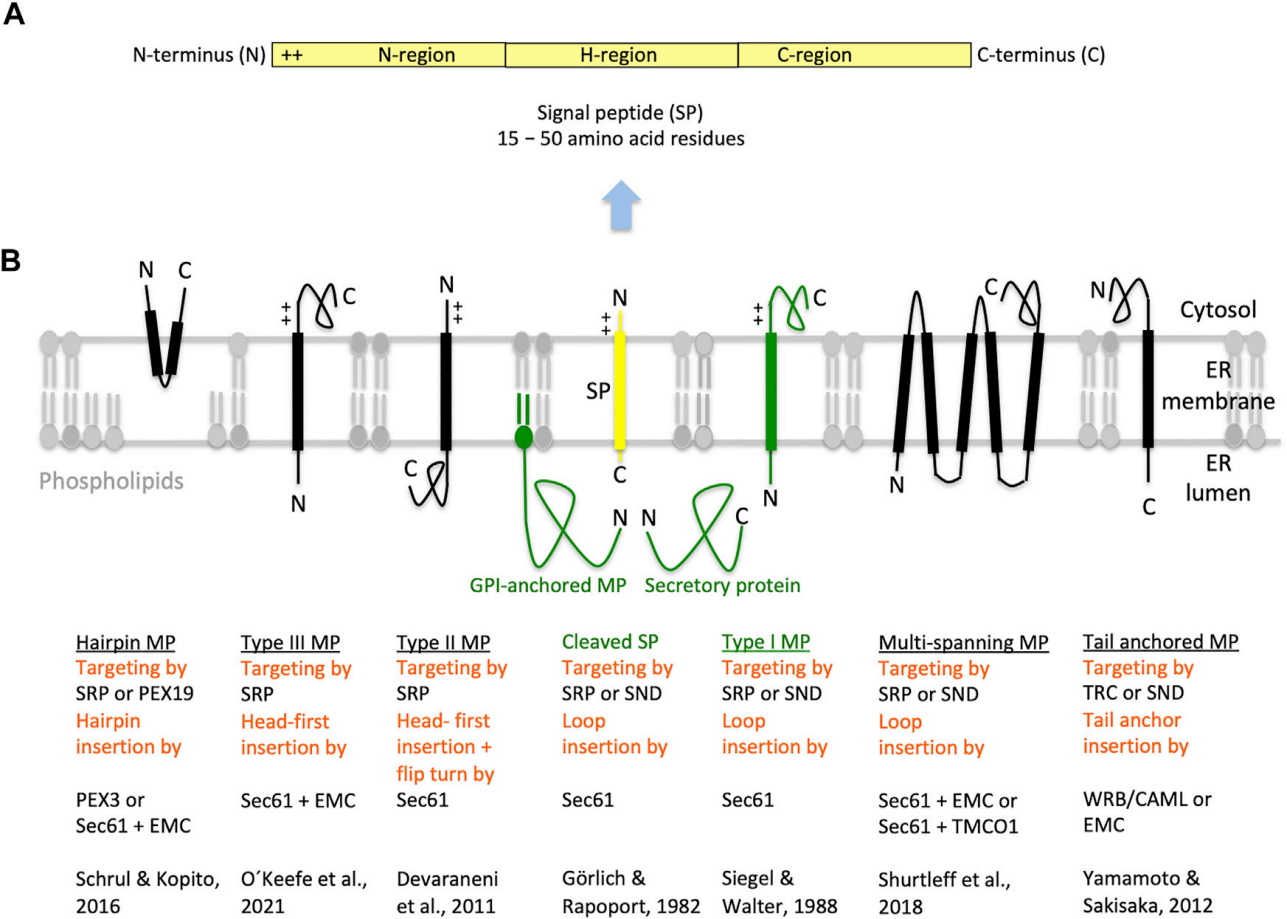
Types of ER membrane proteins and their biogenesis. **(A,B)** The cartoons depict a signal peptide (SP) and six classes of ER membrane proteins (MP, underlined) with their particular membrane protein type and the respective mechanism of ER targeting and membrane insertion (both indicated below the cartoon in red). Cleavable SPs (in yellow) have a tripartite structure and facilitate ER import of secretory proteins (in green), glycosylphosphatidylinositol (GPI)-anchored- and single-spanning type I membrane proteins (in green). In addition, they may mediate ER import of certain multi-spanning membrane proteins, but not of hairpin, single-spanning type II or III, other multi-spanning and TA membrane proteins, which depend on transmembrane helices (TMHs) that serve as SPs and facilitate membrane targeting as well as insertion. Positively charged amino acid residues (+) play an important role in the orientation of membrane proteins and SPs in the membrane; typically, the orientation follows the positive inside rule. In the case of membrane proteins with amino-terminal TMHs, membrane insertion typically involves the same components and mechanisms, which deliver secretory proteins and GPI-anchored membrane proteins to the ER lumen. In certain cases, however, auxiliary membrane protein insertases, such as EMC or TMCO1 complex may be involved. Following their ER import, GPI-anchored membrane proteins become membrane anchored via their carboxy-termini by GPI-attachment. Some key references are given. The Figure and Figure legend were adapted from Sicking et al. (2021a). C, carboxy-terminus; N, amino-terminus.

In case of the SP- or TMH-dependent ER targeting, cotranslational ER targeting of nascent precursor polypeptides and their mRNAs is mediated by the cytosolic signal recognition particle (SRP) and its heterodimeric receptor in the ER membrane, i.e., SRP receptor or SR (Table 1) (Siegel and Walter, 1988; Ng et al., 1996; Egea et al., 2005; Gamerdinger et al., 2015; Hsieh et al., 2020; Jomaa et al., 2021; Meyer and Dobberstein, 1980a; Meyer and Dobberstein, 1980b; Gilmore et al., 1982a; Gilmore et al., 1982b; Tajima et al., 1986). Other binary targeting systems comprising a single ribosome-associating component and a heterodimeric membrane receptor may co- and posttranslationally direct precursor polypeptides to the Sec61 complex and were named TRC-, PEX19/PEX3-, and hSnd2/hSnd3-pathway (Borgese and Fasana, 2011; Yamamoto and Sakisaka, 2012; Borgese et al., 2019; Schrul and Kopito, 2016; Yamamoto and Sakisaka, 2018; Aviram et al., 2016; Casson et al., 2017; Haßdenteufel et al., 2017; Haßdenteufel et al., 2018; Haßdenteufel et al., 2019; Tirincsi et al., 2022b). Some hairpin and most TA membrane proteins depend on dedicated components and posttranslational pathways for their ER targeting and subsequent membrane insertion (Figure 3). The TRC-pathway (termed GET-pathway in yeast) handles TA proteins and the PEX3-dependent pathway some hairpin and certain peroxisomal membrane proteins (Borgese and Fasana, 2011; Yamamoto and Sakisaka, 2012; Borgese et al., 2019; Schrul and Kopito, 2016; Yamamoto and Sakisaka, 2018). In the case of the TRC-pathway, membrane targeting involves the Bag6 complex as well as additional cytosolic factors; in case of the PEX3-dependent pathway, membrane targeting involves cytosolic PEX19 (Table 1). Notably, these pathways are not strictly separated from each other, i.e., there are at least some precursor polypeptides, which can be targeted by more than one pathway. For example, certain small human presecretory proteins with a content of less than 100 amino acid residues, such as preproapelin, can be targeted to the Sec61 complex by the SRP-, SND- as well as the TRC-pathway (Haßdenteufel et al., 2017, 2018, and 2019). Likewise, some TA membrane proteins (such as Sec61ß and RAMP4) can be targeted to the membrane via the same three pathways (Casson et al., 2017). In addition, the Sec61ß coding mRNA can be targeted to the ER by an unknown mechanism (Cui et al., 2015). Thus there is redundancy in the targeting process, i.e., the targeting pathways have overlapping substrate specificities.

SPs for ER protein import, typically, comprise around 25 amino acid residues and have a tripartite structure with a positively charged amino-terminus (defined as N-region), a central hydrophobic region (defined as H-region), and a slightly polar carboxy-terminus (defined as C-region) (Figure 3) (von Heijne, 1985; Hegde and Bernstein, 2006). SPs have a dual function; they target presecretory proteins to the Sec61 complex and trigger the opening of an aqueous channel within the Sec61 complex for translocation of the polypeptide into the ER lumen (Görlich and Rapoport, 1993; Wirth et al., 2003; Dejgaard et al., 2010; Conti et al., 2015; Voorhees and Hegde, 2016). TMH are similar to SP in structure and function, except for the positioning of positively charged amino acid residues, which can be up- or downstream of the central hydrophobic region and determine the TMH orientation in the ER membrane, following the “positive inside rule” (Goder et al., 2004; Baker et al., 2017; Whitely et al., 2021).

### 1.3 Translocation Mechanisms

In human cells, the heterotrimeric Sec61 complex forms a large multicomponent system together with the ribosome and the oligomeric membrane proteins translocon-associated protein (TRAP) and oligosaccharyltransferase (OST), which catalyzes N-linked glycosylation (Figure 1C) (Pfeffer et al., 2012; Pfeffer et al., 2014; Pfeffer et al., 2015; Pfeffer et al., 2017; Mahamid et al., 2016). This super-complex or Sec61 translocon can insert into the membrane or translocate into the lumen a whole variety of topologically very different precursor polypeptides (type I-, type II-, type III-, TA and hairpin membrane proteins and soluble proteins, respectively) (Figure 3). Next, these precursors mature to membrane proteins with one or more hairpins or TMHs, as glycosylphosphatidylinositol- (GPI-) anchored membrane proteins, or soluble proteins in the ER lumen, such as ER-lumenal or secretory proteins (Gemmer and Förster, 2020; O'Keefe et al., 2021a; Tirincsi et al., 2022a; Liaci and Förster, 2021). Typically, membrane insertion and translocation are facilitated by either a cleavable amino-terminal SP or the TMH of the nascent precursor polypeptide, which acts as a non-cleavable SP substitute. Cleavable SPs are removed from the precursor polypeptides in transit by one of the two signal peptidase complexes (SPCs), which have their catalytic sites in the ER lumen (Kalies et al., 1998; Chen et al., 2001; Liaci and Förster, 2021).

Thus, ER protein import involves three stages, i) co- or posttranslational targeting of the precursor to the heterotrimeric Sec61 complex in the ER membrane, ii) head-on (N_ER lumen or out_-C_cytosol or in_) or loop (N_in_-C_out_) insertion of the SP or TMH into the polypeptide-conducting Sec61 channel, and iii) completion of membrane insertion or translocation. Co- and posttranslational insertion of SP or TMH into the Sec61 channel and the simultaneous gating of the Sec61 channel to the open state occur either spontaneously or involve substrate-specific auxiliary components of the Sec61 channel (such as TRAP, Sec62/Sec63, TRAM1) (Wiedmann et al., 1987; Fons et al., 2003; Menetret et al., 2008; Sommer et al., 2013; Lang et al., 2012; Lakkaraju et al., 2012; Ziska et al., 2019; Görlich et al., 1992a; Voigt et al., 1996; Hegde et al., 1998; Sauri et al., 2007). Typically, the orientation of SP- and TMH in the Sec61 channel follows the positive inside rule (Goder et al., 2004; Baker et al., 2017), i.e. positively charged amino acid residues in the N-region support loop insertion (N_in_-C_out_) and positively charged residues downstream of the SP or TMH interfere with loop insertion and, therefore, favour head-on insertion (N_out_-C_in_) that can be followed by a “flip turn” (Figure 3) (Devaraneni et el., 2011).

Following the pioneering work by Blobel and Dobberstein (1975a) and Blobel and Dobberstein (1975b), ER protein import was studied in cell-free assays, which involve synthesis of a single precursor polypeptide in the presence of ER derived membrane vesicles or proteoliposomes and allow the conclusion of whether and how targeting and membrane insertion or translocation of a certain precursor can be facilitated by a certain component. Recently, more global approaches were employed, such as proximity-specific ribosome-profiling (Reid and Nicchitta, 2012; Calvin et al., 2014; Hsu et al., 2018; Shurtleff et al., 2018; Hannigan et al., 2020) and quantitative proteomics (Nguyen et al., 2018; Shurtleff et al., 2018; Tian et al., 2019; Klein et al., 2020; Schorr et al., 2020; Bhadra et al., 2021a; Zimmermann et al., 2021; Tirincsi et al., 2022b). We started to address the question which precursors use a certain pathway or component in intact human cells, i.e., under conditions of fast translation rates and in the presence of competing precursors. Typically, our approach employed siRNA-mediated depletion of single components in HeLa cells, label-free quantitative proteomic analysis, and differential protein abundance analysis to characterize client specificities of various components.

## 2 Summary of Previously Reported Results From Label-Free Proteomics

### 2.1 A Proteomic Approach for the Analysis of Protein Import Into the Human Endoplasmic Reticulum

**FIGURE 4:**
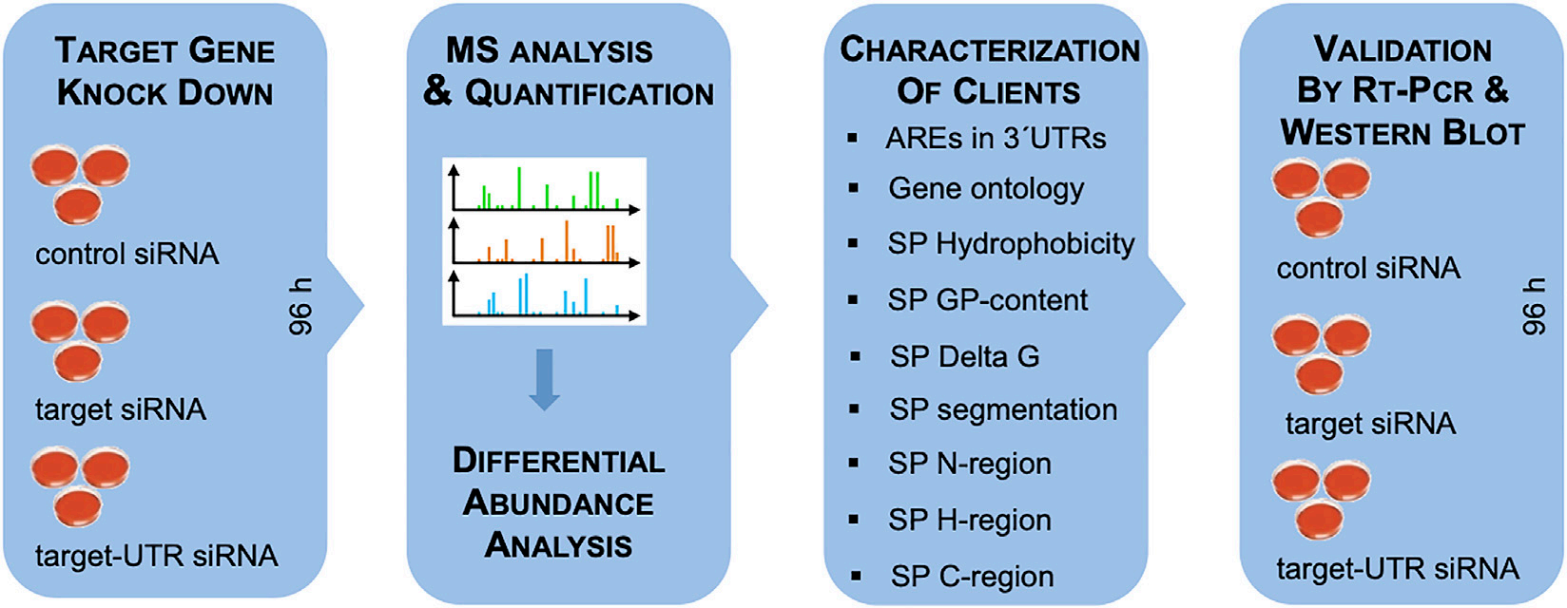
Experimental strategy. The experimental strategy involved i) siRNA-mediated gene silencing using two different siRNAs for each target and one non-targeting (control) siRNA, respectively, with three replicates for each siRNA for 96 h; ii) label-free quantitative analysis of the total cellular proteome; iii) differential protein abundance analysis to identify negatively affected proteins (i.e., putative substrates or clients of the target) and positively affected proteins (i.e., putative compensatory mechanisms); iv) independent validation by western blot. For characterization of substrates, genes were screened for AU-rich elements (ATTTA motifs) in 3′UTRs using the AREsite2 database (http://rna.tbi.univie.ac.at/AREsite2/welcome) and SPs were analyzed for hydrophobicity according to Kyte and Doolittle, (1982) (https://www.bioinformatics.org/sms2/protein_gravy.html), GP-content (Nguyen et al., 2018), apparent Delta G for membrane insertion of TMHs (http://dgpred.cbr.su.se), or segmentation (https://phobius.sbc.su.se) in combination with characterization of the SP segments with the same tools as above.

Our experimental approach was designed to identify substrates or clients of components, which are involved in targeting or translocation of precursor polypeptides into the human ER under cellular conditions, thereby setting it apart from experiments where single precursor proteins are studied one by one in either cell-free systems for synthesis of proteins and their import into ER-derived vesicles (rough microsomes) or proteoliposomes, or the ER of semi-permeabilized cells, or under conditions where single precursor proteins are over-produced in cells. The approach represents a combination of siRNA-mediated knock down or CRISPR/Cas9-mediated knock-out of a certain protein targeting or translocation component in human cells (such as HeLa or HEK293 cells), label-free quantitative mass spectrometric (MS) analysis of the total cellular proteome, and differential protein abundance analysis for two different cell pools that had been treated with two different siRNAs, which target the same mRNA, compared to a pool of cells, which had been treated with a non-targeting siRNA (defined as negative control) (Figure 4). In the case of knock-out cells, only two cell pools were compared, a control cell line and the knock-out line; where available, deficient patient fibroblasts were analyzed (as in the case of Congenital disorders of glycosylation or Zellweger syndrome). The approach is based on the expectations that precursors polypeptides, destined to the ER, are degraded by the cytosolic proteasome upon interference with their ER import. Therefore, their cellular levels decrease compared to control cells, which is detected by quantitative MS in combination with subsequent differential protein abundance analysis (Figure 4). In several depletions (SEC61A, TRAPB, WRB, SRA) the absence of the target subunit, typically, caused degradation of the other subunit(s) of the complex or even other components of the same pathway, reminiscent of the “use it or loose it principle” of muscle physiology (Nguyen et al., 2018; Tirincsi et al., 2022b). Consistent with the starting expectation, the decrease of secretory pathway proteins was accompanied by an increase in ubiquitin-conjugating enzymes in the cytosol. Furthermore, in some cases the concomitant increase in other ER import components was observed, which may point to a possible functional, compensatory overlap between different pathways. Alternatively or additionally, we observed an increase in components for protein import into mitochondria, which appears to be an alternative to protein degradation in preventing aggregation of potentially dangerous polypeptides in the cytosol (Pfeiffer et al., 2013). All these phenomena were inversely correlated with the severity of the negative effect on secretory pathway proteins.

As a proof-of-principle, the approach was established for the Sec61 complex, which is necessary for or at least involved in the ER import of most precursor polypeptides (Figures 1–3; Supplementary Table S1) (Nguyen et al., 2018). In general, the timing of the experiment was optimized to seeding of the cells on day one with two consecutive siRNA transfections on the same and the following day and harvesting of the cells on day four (Figure 4). Alternatively, CRISPR/Cas9-treated knock-out cells and, in some cases, deficient patient fibroblasts were cultivated in parallel to the respective control cells for 96 h.

Typically, between 5,000 and 6,500 different proteins were quantified and statistically analyzed (Supplementary Tables S1, S2), including proteins with low and high cellular concentrations, which ranged from below 1 to almost 10,000 nM (Hein et al., 2015; Schorr et al., 2020; Bhadra et al., 2021a). For the control cells, Gene Ontology (GO) terms assigned the expected 26%–29% of proteins to organelles of the endocytic and exocytic pathways plus the extracellular space and plasma membrane (Supplementary Tables S1, S2). In the case of depletion or deficiency of an ER targeting or translocation component, GO terms assigned between 35% and 60% of the negatively affected proteins to organelles of the pathways of endocytosis and exocytosis plus cell surface, representing a more or less pronounced enrichment as compared to the total quantified proteome (Supplementary Tables S1, S2). Furthermore, similar enrichment of precursor proteins with SP, N-glycosylation, or membrane location was typically detected, and cytosolic proteins were under-represented among the negatively affected proteins (with the exception of KTN1 depletion, see below). Taken together, these results indicated that the precursors of these negatively proteins are substrates or clients of the respective component of interest.

As stated above, 30% of the total quantified proteome comprises ER protein import substrates. However, even in the case of Sec61 depletion, only 197 proteins with SP plus 98 with TMH, i.e., about 300 of the 6,000 quantified proteins or 5%, were negatively affected by the depletion (Supplementary Table S1). Thus, our experimental approach underestimates the number of different precursor polypeptides relying on this component by far. As expected, the numbers of negatively affected proteins were even lower for all the other translocation and targeting components since these components are expected to be precursor-specific, i.e., involved in import of only certain precursor polypeptides (Nguyen et al., 2018; Tian et al., 2019; Klein et al., 2020; Schorr et al., 2020; Bhadra et al., 2021a; Zimmermann et al., 2021; Tirincsi et al., 2022b). Obviously, this raises the question why we see only the tip of the iceberg in respect to clients. There are several contributing factors under conditions of siRNA-mediated knock-down: i) The depletion efficiency and its duration, which were optimized for minimal effects on cell growth and viability, was not high enough to cause significant accumulation and degradation of precursor proteins. Typically, the MS data suggested a depletion of close to 90% for the targeting or translocation component, which was confirmed by the validating western blot analysis. Thus the residual amount of the component of interest may have been sufficient to sustain the physiological functions of depleted proteins over the duration of the experiment. ii) As stated above, a certain function in ER protein import in human cells is compensated by other proteins or pathways. Except for the Sec61 complex, we actually expected that to be the case. iii) Some client proteins may have remained largely unaffected because they either have longer half-lives than the component of interest or may have a higher than average affinity for the component of interest. iv) Last but not least, some accumulating precursors may have stayed soluble in the cytosol, aggregated, or ended up in mitochondria where they were protected from degradation by the proteasome. Notably, we have observed mistargeting of certain precursors of secretory proteins into mitochondria in the absence of Sec61 function in human cells (Pfeiffer et al., 2013). Under knock-out conditions, the cells may also have adapted to the absence of a certain component, a phenomenon we observed to a certain extent even under siRNA-mediated depletion conditions in form of positively affected transport components (Nguyen et al., 2018; Tian et al., 2019; Klein et al., 2020; Schorr et al., 2020; Bhadra et al., 2021a; Zimmermann et al., 2021; Tirincsi et al., 2022b).

### 2.2 mRNA Targeting to the Human Endoplasmic Reticulum

**FIGURE 5:**
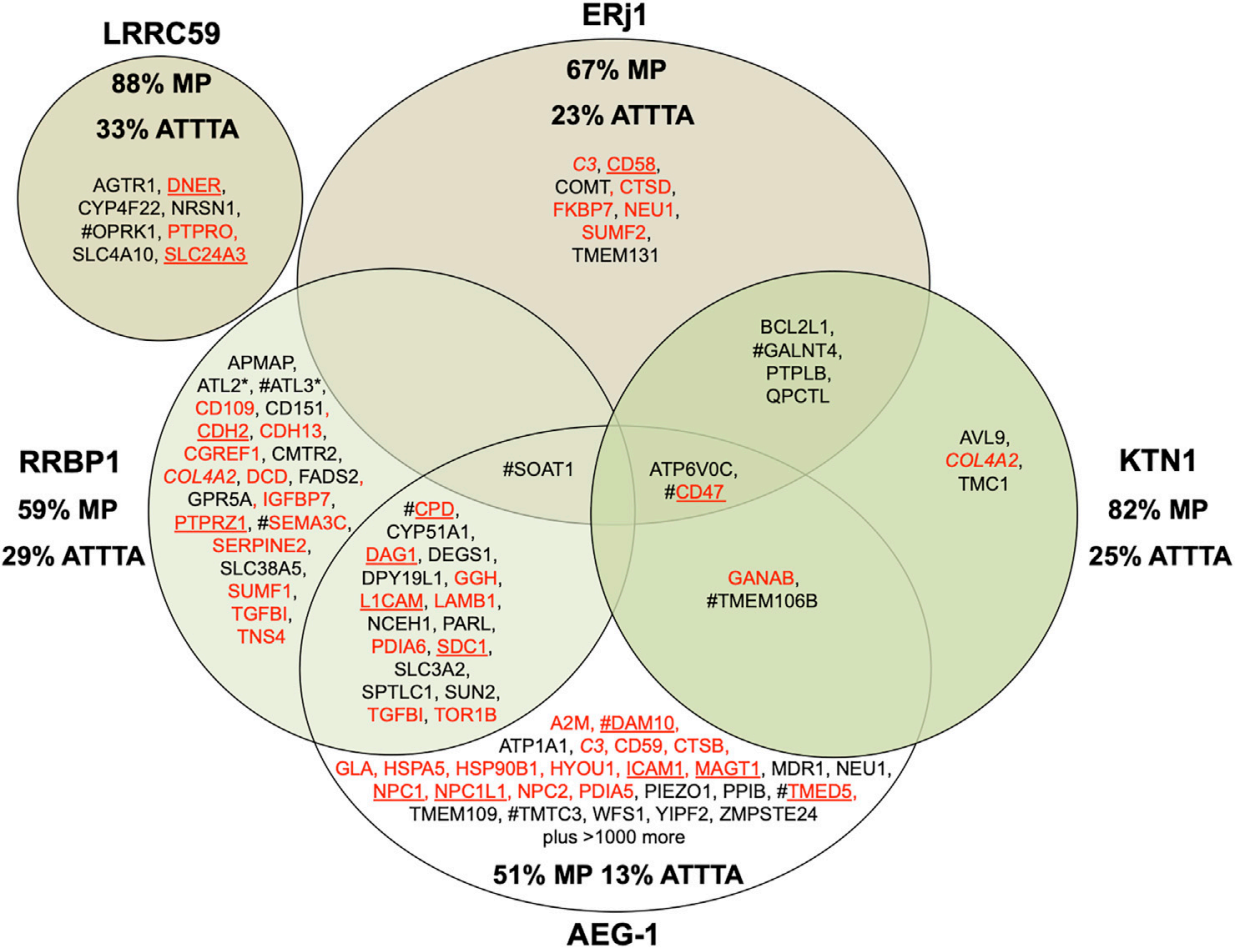
Venn diagram for putative mRNA and RNC targeting components in the human ER membrane and their clients. The diagram summarizes precursors with SP (in red) or TMH (in black), which are destined to the secretory pathway and were negatively affected by the indicated depletion in HeLa cells (Bhadra et al., 2021) or were identified by mRNA crosslinking and ribosome proximity labeling, respectively (Hsu et al., 2018; Hannigan et al., 2020); membrane protein precursors with SP are underlined. All these precursor polypeptides are defined as potential clients or substrates of the respective component. The percentage of membrane proteins (MP) of the secretory pathway among the respective clients is indicated (%). For AEG-1 randomly selected examples of secretory pathway clients are shown and the % MP refers to the complete set of clients; for LRRC59 the secretory pathway representatives among the top twenty clients are shown and the % MP refers to these clients. COL4A2 is in italics to highlight that it was negatively affected by RRBP1 as well as KTN1 depletion. # highlights the presence of multiple ATTTA motifs in all mRNAs clients (i.e., ATTTA ≥ 10), which was tested for the top twenty clients in the case LRRC59 and for the complete set of clients in all other cases. Asterisks indicate hairpin proteins.

As stated in the Introduction, there is SP- and, therefore, SRP-independent targeting of mRNAs or ribosome nascent chain complexes (RNCs) to the ER (Figure 2). According to pioneering biochemical and cell biological analysis by C. Nicchitta and coworkers, the synthesis of various types of polypeptides, such as cytosolic proteins, is initiated on 80S ribosomes or even 60S ribosomal subunits, which remain associated with the ER after termination of protein synthesis (Seiser and Nicchitta, 2000; Potter at al., 2001; Pyhtila et al., 2008; Reid and Nicchitta, 2012). As of today, the involved mRNA targeting appears to involve mRNA receptor proteins in the ER membrane, i.e., AEG-1 (Hsu et al., 2018), LRRC59 (Tazawa et al., 1991; Ohsumi et al., 1993; Hannigan et al., 2020), RRBP1 (Savitz and Meyer, 1990 and 1993; Bhadra et al., 2021a) and KTN1 1 (Ong et al., 2003; Ong et al., 2006; Bhadra et al., 2021a) (Table 1). Proximity-specific ribosome-profiling experiments, however, suggested ER-targeting of RNCs with nascent polypeptide chains, which are not sufficiently long to interact with SRP, play a more important role in mRNA targeting to the ER than direct targeting of mRNA to ER-associated ribosomes (Calvin et al., 2014). Notably, the first is translation-dependent, the latter is translation-independent (Figure 2). Insights into the possible specificities of these mRNA targeting reactions, however, are only beginning to accumulate (Hsu et al., 2018; Hannigan et al., 2020; Bhadra et al., 2021a). Until recently, there were just a couple of precursor polypeptides known to involve RRBP1 either as receptor for RNCs or mRNA, i.e., the SP-comprising precursors of the GPI-anchored membrane protein placental alkaline phosphatase (Cui et al., 2012; Cui et al., 2013), of certain Collagens (i.e., collagens Iα1 plus Iα2 and IIIγ) (Ueno et al., 2010) and of the ER-resident protein Calreticulin (Cui et al., 2012). More recent global data from mRNA crosslinking or ribosome proximity labeling in combination with transcriptome analysis, however, gave first glimpses of the substrate spectra of the two mRNA receptors AEG-1 (Hsu et al., 2018) and LRRC59 (Hannigan et al., 2020) (Figure 5).

We employed our MS approach to identify precursor polypeptides that may involve targeting of the corresponding mRNAs or RNCs by the two putative mRNA- or RNC-receptors RRBP1 and KTN1 (Bhadra et al., 2021a). The approach suggested an additional collagen (i.e., COL4A2) and two ER-resident hairpin membrane proteins (i.e., ATL2 and ATL3) among the 39 negatively affected proteins as RRBP1 clients (Figure 5; Supplementary Table S1). For RRBP1, a role as RNC-receptor in the biogenesis of 22 precursors with SP (including six membrane protein precursors) plus 17 precursors with TMH, destined to the secretory pathway, was also supported by the positive effect on SRA and SRB as a consequence of RRBP1 depletion. For KTN1, in contrast, it turned out that not only proteins, destined to the ER (i.e., three precursors with cleavable SP –including one membrane protein- plus eight precursors with TMH), are degraded in the absence of KTN1 but also several cytosolic proteins, most notably cytoskeletal proteins and protein kinases (Supplementary Table S1, see below). The negative effect on ER protein import, however, is consistent with the idea that KTN1 can also play a role in the biogenesis of proteins, destined to the secretory pathway, as was suggested by the negative effect on the membrane protein precursor CD47 as well as the positive effect on RRBP1 (Figure 5). This view is consistent with our observation that crosslinking of native human microsomes and subsequent MS analysis observed among several intra-molecular crosslinks for KTN1 the intermolecular crosslink (peptide _42_REQKLIPTK_52_) to the translocon subunit TRAPγ (peptide _82_FVLKHK_89_) (Fan, L. and Jung, M., unpublished). Furthermore, the negative effect on CD47 suggested a function of KTN1 as the elusive ER-resident mRNA receptor in the so-called TIGER domain, which was proposed by C. Mayr to form a cytosolic micro-domain, which allows the enrichment of membrane protein-encoding mRNAs with multiple AU-rich elements (AREs, specifically ATTTA motifs) in their 3′ UTRs in the ER vicinity (Berkovits and Mayr, 2015; Ma and Mayr, 2018) (Figure 2). Thus, the key observation may be that KTN1 plays a role in targeting of certain mRNAs to ER subdomains. In the case of ERj1 (Dudek et al., 2002; Dudek et al., 2005; Blau et al., 2005; Benedix et al., 2010), another ER membrane protein that was proposed to interact with mRNAs or RNCs, the proteomic approach supports a function in cotranslational ER protein import rather than in ER targeting of mRNAs or RNCs (see below). Interestingly, when we compared our results with the published results from ribosome profiling experiments for the other two mRNA targeting components, we noticed that AEG-1 showed considerable overlap with clients of both KTN1 and RRBP1 while there was no overlap detected for LRRC59 (Figure 5) (Hsu et al., 2018; Hannigan et al., 2020). When the different putative clients were analyzed for ATTTA motifs, there were no general rules for mRNA recognition by these receptors emerging from the available data, with the possible exception of these motifs in the case of some KTN1- and RRBP1-clients (Bhadra et al., 2021a). The authors found that multiple ATTTA motifs (≥10) are present in the 3′ UTRs of mRNA clients of the different receptors to varying degrees, ranging from 13% to 33% (Figure 5). Thus, different motifs in the mRNAs appear to play a role.

In striking contrast to all other depletions of proteins that are involved in ER protein import, KTN1 depletion affected predominantly cytosolic proteins, i.e., their level increased from the average of 29% to 39%. In total, 21 cytosolic proteins were negatively affected, including two metabolic enzymes (GAPDH and GAPDHS), several protein kinases (OXSR1, PAK1, PDPK1, PDPK2 and ZAK), and various cytoskeletal proteins (Junction Plakoglobin, Myosin 11, Vinculin and Gamma-tubulin complex component 4). This raises the interesting question why cytosolic proteins should be degraded after their synthesis on free cytosolic ribosomes. We hypothesize that for the negatively affected cytoskeletal proteins it may be of importance to be synthesized and sequestered near their site of action rather than distributed throughout the cytosol, in particular for membrane interacting cytoskeletal proteins, such as Junction Plakoglobin and Vinculin, at adherens junctions between neighboring cells. In analogy, this may be true for certain protein kinases, such as OXSR1 (which is involved in regulating the actin cytoskeleton in response to environmental stress), PAK1 (which regulates cytoskeletal reorganization for cell motility and morphology), and PDPKs 1 and 2 (which are also located at cell junctions). Notably, multiple ATTTA motifs were also detected in the mRNAs of several cytosolic KTN1 clients (16% as compared to 31% of cytosolic clients of RRBP1, 43% for LRRC59, 13% for AEG-1, and 21% for ERj1) (Bhadra et al., 2021a).

On the basis of the data it was proposed that KTN1 may represent the mRNA-binding protein that resides in the ER membrane and is enriched in the TIGER domain in order to take over mRNAs from the cytosolic RNA-binding TIS11B and allow initiation of their translation by Sec61-associated ribosomes (Figure 2). If the mRNA codes for a membrane protein precursor with SP (such as CD47) or with an amino-terminal TMH, the nascent precursor begins to sample the Sec61 channel, which leads to spontaneous channel opening or the recruitment of auxiliary factors of the Sec61 channel. Since ERj1 was found to have overlapping substrate specificities with KTN1 in our proteomic studies, we suggest it to cooperate with KTN1 in allowing Sec61 channel opening when BiP is bound to ERj1′s J-domain (Dudek et al., 2002; Blau et al., 2005; Dudek et al., 2005; Benedix et al., 2010; Schäuble et al., 2012). Subsequently, the precursor is translocated into the ER or integrated into the ER membrane (CD47). If the mRNA codes for a cytosolic protein, however, sampling of the Sec61 channel remains unproductive and NAC gets access to the amino-terminus of the nascent polypeptide and causes its release from Sec61 and the concomitant release of the ribosome from Sec61 (Moeller et al., 1998). Next, synthesis of the cytosolic protein is completed and the protein is enriched in the TIGER domain to play its physiological role (Berkovits and Mayr, 2015; Ma and Mayr, 2018).

### 2.3 Precursor Polypeptide Targeting to the Human Endoplasmic Reticulum

#### 2.3.1 SRP/SR

The signal hypothesis for targeting of nascent precursor polypeptides to the ER was put forward by G. Blobel et al. (Blobel, 1980). In later versions, it proposed that the amino-terminal SP of a nascent presecretory protein is recognized and bound by cytosolic SRP, which mediates a translational attenuation and facilitates association of the RNC-SRP complex with the heterodimeric SRP receptor (SR), which is membrane-anchored via the β-subunit (Siegel and Walter, 1988; Ng et al., 1996; Egea et al., 2005; Gamerdinger et al., 2015; Hsieh et al., 2020; Meyer and Dobberstein, 1980a; Meyer and Dobberstein, 1980b; Gilmore et al., 1982a; Gilmore et al., 1982b; Tajima et al., 1986; Jomaa et al., 2021; Jomaa et al., 2022). The interaction of SRP with SR drives the mutual hydrolysis of bound GTP and leads to transfer of the RNC to the Sec61 complex (Halic and Beckmann, 2005, Halic et al., 2006; Jomaa et al., 2021). Thus, SRP represents a precursor as well as a mRNA targeting device (Figure 2). Comparative ribosome profiling experiments addressed functionality of the bacterial and yeast SRP *in vivo* (Chartron et al., 2016; Schibich et al., 2016; Costa et al., 2018) and demonstrated the strong preference of SRP for TMHs regardless of their position relative to the amino-terminus of the nascent polypeptide chain. Furthermore, they demonstrated the efficient ER targeting of precursors with just cleavable SPs in absence of SRP. Thereby, these studies stretch the versatility of SRP and reconciled two important considerations. First, the comparatively low abundance of SRP as compared to the abundance of translating ribosomes can be compensated by an mRNA targeting step, probably extending the time-window for the target recognition by SRP. Second, the crowded environment at the ribosomal tunnel exit can be eased by multiple iterations for SRP recognition without being limited to recognition of the SP or first TMH.

Late in the 1980s, characterization of precursor proteins with the ability for SRP-independent ER targeting, such as small presecretory proteins in mammalian cells and TA-membrane proteins in mammalian and yeast cells suggested alternative ER targeting machineries (Müller and Zimmermann, 1987; Schlenstedt and Zimmermann, 1987; Schlenstedt et al., 1990; Kutay et al., 1993; Ast et al., 2013). In the early 2000s, some small model presecretory proteins were shown to be targeted to the mammalian ER membrane in an SRP-independent fashion by their interaction with the cytosolic protein calcium-calmodulin and its putative association with the calcium-calmodulin (Ca^2+^-CaM)-binding site in the cytosolic amino-terminus of the Sec61α protein, possibly representing yet another targeting mechanism (Shao and Hegde, 2011; Schäuble et al., 2012). With respect to pathway interconnections, it is interesting to note that Ca^2+^-CaM was found to inhibit rather than stimulate targeting of TA proteins to the ER membrane (Haßdenteufel et al., 2011).

**FIGURE 6:**
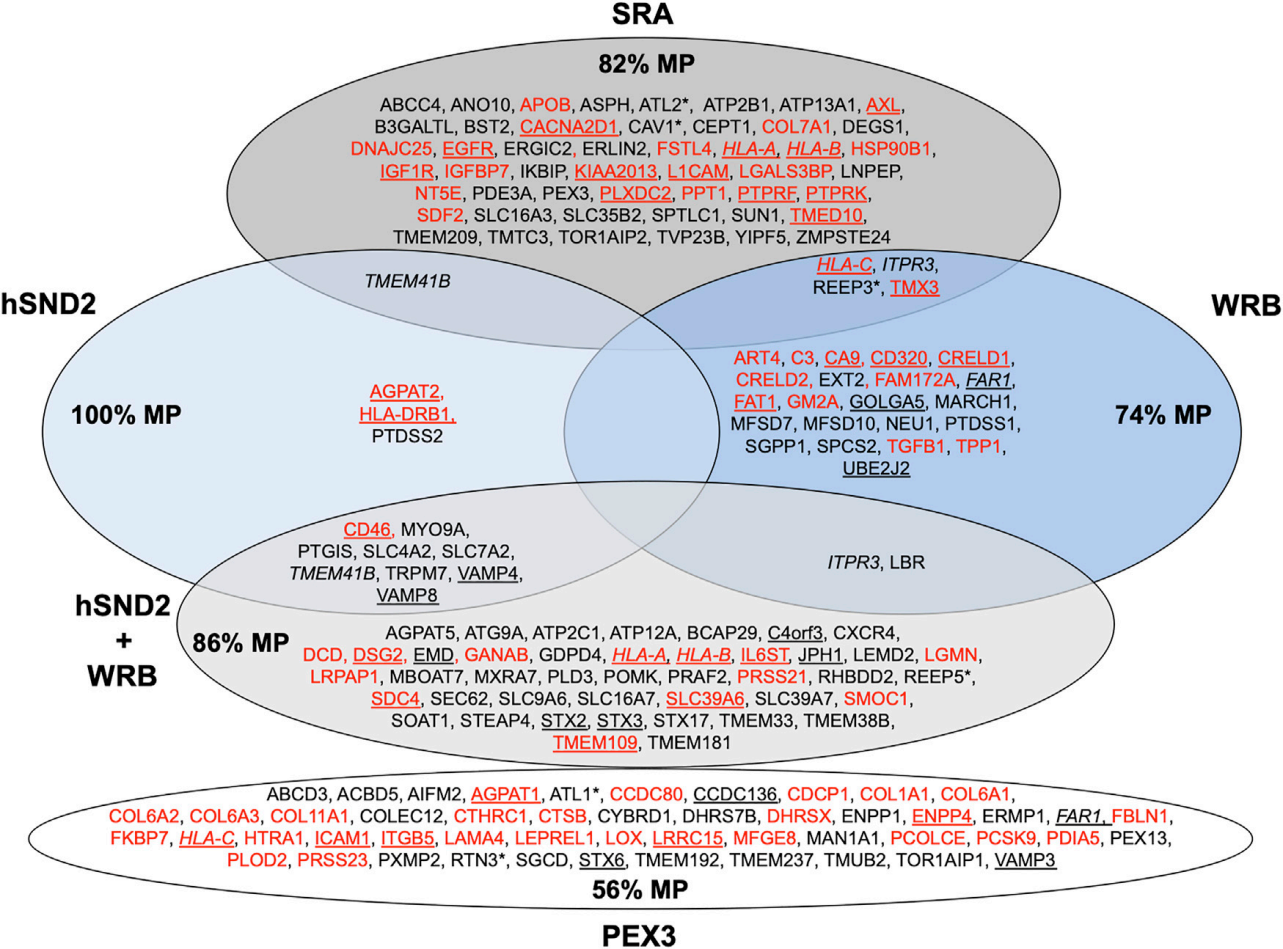
Venn diagram for components for precursor targeting to the human ER membrane and their clients. The diagram summarizes precursors with SP (in red) or TMH (in black), which are destined to the secretory pathway and were negatively affected by the indicated depletion in HeLa cells (Tirincsi et al., 2022b) or by PEX3 depletion in HeLa cells and after PEX3 knock-out in Zellweger patient fibroblasts (Zimmermann et al., 2021); membrane protein precursors with SP are underlined, as are TMH proteins with TA. All these precursor polypeptides are defined as potential clients or substrates of the respective component or complex. The percentage of membrane proteins (MP) of the secretory pathway among the respective clients is indicated (%). HLA-C is in italics to highlight that it was negatively affected by PEX3 as well as SRA and WRB depletion, likewise are highlighted FAR1 and HLA-A, HLA-B, ITPR3 plus TMEM41B, respectively. Asterisks indicate hairpin proteins.

Recently, we applied the proteomic strategy to identify precursor polypeptides that depend on SR for their targeting to the ER. Applying the established statistical analysis, we found that SRA depletion significantly affected the steady-state levels of 139 proteins: 133 negatively and 6 positively (Figure 6; Supplementary Table S1) (Tirincsi et al., 2022b). Among the negatively affected proteins, GO terms assigned 50% to organelles of the endocytic and exocytic pathways, thus representing a firm enrichment compared to the total quantified proteome (26%). Furthermore, we detected significant enrichment of precursor proteins with SP, N-glycosylated proteins, and membrane proteins. The negatively affected proteins included 24 proteins with cleavable SP, among them 14 membrane proteins plus 30 membrane proteins with TMH, including the ER hairpin membrane protein ATL2, many single-spanning membrane proteins and several multi-spanning membrane proteins, including the hairpin protein REEP3. Thus, the precursors of these negatively affected proteins with SP and TMH can be expected to be clients of the SRP and SR targeting pathway. When the SPs of SR-dependent precursor polypeptides were analyzed for hydrophobicity, GP content, and SP segmentation no significant distinguishing features were determined. Overall, SRP and SR clients showed a preference for cleavable SP (44%) or non-cleavable N-terminal targeting signals (77% of the remaining membrane protein clients) and an underrepresentation of TA, which is consistent with previous results from proximity-based ribosome profiling experiments (Chartron et al., 2016; Costa et al., 2018).

#### 2.3.2 TRC

TA proteins are defined as single-spanning membrane proteins with a defining carboxy-terminal TMH (Kutay et al., 1993). Approximately 1% of the human protein-coding genome code for TA proteins. Not all of these, however, have a functional association with the secretory pathway (Borgese and Fasana, 2011; Borgese et al., 2019). TA proteins of the secretory pathway, such as the β- and γ-subunits of the Sec61 complex, the redox protein Cytochrome b_5_, many apoptosis-associated proteins (including various Bcl family members) and many vesicular trafficking components (i.e., Syntaxins and VAMPs), have to be targeted to and inserted into the ER membrane (Borgese and Fasana, 2011; Borgese et al., 2019). Similar to SRP-mediated targeting, TA proteins are directed to the ER membrane via a heterodimeric ER membrane resident receptor complex, made up by WRB and CAML. The minimal cytosolic targeting machinery for TA proteins was termed TA receptor complex (TRC) in mammalian cells (Table 1). The cytosolic ATPase TRC40 binds the TA protein with its hydrophobic binding pocket and the WRB/CAML complex facilitates their efficient ER targeting. The WRB/CAML complex also facilitates the actual membrane insertion (Schuldiner et al., 2008; Yamamoto and Sakisaka, 2012; Borgese et al., 2019). Additionally, the TA targeting machinery includes a ribosome binding complex (comprising Bag6, Ubl4A, and TRC35), which acts upstream of TRC40 (Leznicki et al., 2010; Mariappan et al., 2010; Leznicki et al., 2011).

We applied our experimental strategy to identify precursor polypeptides that depend on WRB for their ER targeting (Tirincsi et al., 2022b). Applying the established statistical analysis, we found that WRB depletion significantly affected the steady-state levels of 296 proteins: 144 negatively and 152 positively (Figure 6; Supplementary Table S1). Among the negatively affected proteins, GO terms assigned 45% to organelles of the pathways of endocytosis and exocytosis. Some enrichment of precursor proteins with SP and membrane proteins was also detected. The identified precursors included 13 proteins with cleavable SP (including six membrane proteins) and 14 membrane proteins without SP, including the ER hairpin protein REEP3. When the SPs of WRB-dependent precursor polypeptides were analyzed for hydrophobicity, GP content, and SP segmentation, no significant distinguishing features were determined. However, when more WRB clients were identified under conditions of simultaneous depletion of WRB and hSnd2 (Figure 6) a preference of WRB for multispanning membrane proteins became visible and more WRB membrane protein clients were observed to have relatively more central and more carboxy-terminal TMH as compared to SRA dependent membrane proteins (Tirincsi et al., 2022b). Taken together, these results on the client spectrum of WRB point towards a more general targeting role of the TRC pathway than previously anticipated and may explain why pathogenic variants of TRC35 or TRC40 as well as CAML are linked to Congenital disorders of glycosylation in humans (Wilson et al., 2022). Notably, first hints towards this end already came from previous reports that small human presecretory proteins can be targeted to the ER of semi-permeabilized human cells by SR, WRB and hSnd2 (Haßdenteufel et al., 2018; Haßdenteufel et al., 2019) and that the cytosolic TRC pathway-component SGTA, which works upstream of Bag6, Ubl4A, and TRC35, is cotranslationally recruited to ribosomes, which synthesize a diverse range of membrane proteins, including those with cleavable SP (Leznicki and High, 2020).

#### 2.3.3 SND

Although roughly one dozen genes coding for yeast TA proteins were characterized as essential, knock-out strains for the TA targeting components are viable, suggesting at least one further targeting route (Schuldiner et al., 2008). Indeed, in 2016 a high-throughput screening approach in yeast by M. Schuldiner and coworkers identified a novel targeting pathway, termed SRP-independent (SND) (Aviram et al., 2016). Three components of this pathway were identified and named Snd1, Snd2, and Snd3 (Table 1). Two hallmarks of the SND targeting pathway emerged. First, similar to the SRP and TA targeting pathways, precursor polypeptides were targeted via the combination of a cytosolic factor (named Snd1) and a heterodimeric receptor in the ER membrane (termed Snd2 and Snd3). Previously, Snd1 had already been described as a ribosome-binding protein. Second, the SND pathway showed a preference for substrates with a central, rather than an amino- or a carboxy-terminal TMH. Furthermore, the SND pathway was able to provide an alternative targeting route for clients with a TMH at their amino- or carboxy-terminus (Aviram et al., 2016). Subsequent sequence comparisons identified the ER membrane protein TMEM208 as putative human Snd2 ortholog (named hSnd2) (Aviram et al., 2016). In experiments, combining siRNA-mediated gene silencing with protein transport into the ER of semi-permeabilized human cells in cell-free assays, hSnd2 appeared to have a similar function as its yeast ortholog (Casson et al., 2017; Haßdenteufel et al., 2017; Haßdenteufel et al., 2018), i.e., the TA membrane protein Cytochrome b_5_ as well as some small presecretory proteins were targeted to the Sec61 complex in the mammalian cell-free assay. Briefly, the human hormone precursor proteins preproapelin and prestatherin can use Sec62 as well as SR for ER targeting in the cell-free assay. Interestingly, prestatherin preferred SRα over Sec62-mediated targeting, whereas preproapelin did the opposite, which was attributed to the higher hydrophobicity of the prestatherin SP (apparent ΔG −0.91 versus −0.19). Taken together with the observation that carboxy-terminal extension (by 187 amino acid residues) of preproapelin or prestatherin by the cytosolic protein DHFR leads to Sec62 independence, our data support the notion that small presecretory proteins use the SRP pathway for Sec61 targeting in human cells inefficiently, because the corresponding nascent chains are prone to be released from ribosomes before SRP can interact (Müller and Zimmermann, 1987; Schlenstedt and Zimmermann, 1987; Müller and Zimmermann, 1988; Schlenstedt et al., 1990; Lakkaraju et al., 2012; Haßdenteufel et al., 2018). Therefore, these precursors rely on alternative targeting pathways. In addition to SR and Sec62, co- and posttranslational targeting of preproapelin and prestatherin can also involve the TRC- and the SND-pathway, albeit with different efficiencies (Haßdenteufel et al., 2018). An ortholog of Snd1 has not yet been characterized in human cells. Recently, precursors of TRPC6 and various GPI-anchored proteins, such as CD55, CD59, and CD109 were added to the growing list of SND-clients (Talbot et al., 2019; Yang et al., 2021).

We applied the established proteomic strategy to identify precursor polypeptides that depend on hSnd2 for their targeting to the ER (Tirincsi et al., 2022b). Applying the established statistical analysis, we found that transient and partial hSnd2 depletion significantly affected the steady-state levels of 76 proteins: 43 negatively and 33 positively (Figure 6; Supplementary Table S1). Among the negatively affected proteins, GO terms assigned roughly 47% to organelles of the endocytic and exocytic pathways. We also detected a small enrichment of N-glycosylated proteins and a large one of membrane proteins. The negatively affected proteins included three proteins with cleavable SP (all being membrane proteins), and nine membrane proteins with TMH, including TA membrane proteins (such as Cytochrome b_5_) plus single-spanning and multi-spanning membrane proteins (such as TRPM7), thus confirming previously observed classes of hSnd2 clients (TRPC6, Cytochrome b_5_). Thus, there seems to be a preference of the human SND system for membrane protein precursors (Figure 6). When the SPs of hSnd2-dependent precursor polypeptides were analyzed for hydrophobicity, GP content, and segmentation, no significant distinguishing features were determined. However, when more hSnd2 clients were identified under conditions of simultaneous depletion of hSnd2 and WRB (Figure 6) a preference of hSnd2 for multispanning membrane proteins became visible and more hSnd2 membrane protein clients were found to have relatively more central or carboxy-terminal TMHs than SRA dependent membrane proteins (Tirincsi et al., 2022b), the latter two aspects being consistent with results for the yeast SND targeting pathway (Aviram et al., 2016). These observations are consistent with the fact that only little overlap between SRA clients and clients of the SND and TRC pathways was detected (Figure 6) and may explain why the latter two pathways can partially substitute for each other. In addition to SND clients, simultaneous depletion of hSnd2 and WRB negatively affected the ER membrane protein TMEM109, which was subsequently characterized as the hitherto elusive hSnd3 in experiments that were addressing its interaction with hSnd2 as well as its role in ER protein import (Tirincsi et al., 2022b).

#### 2.3.4 PEX19/PEX3

Furthermore, recent work characterized the PEX19/PEX3-dependent pathway as a fourth pathway for targeting of precursor polypeptides to the ER (Schrul and Kopito, 2016; Yamamoto and Sakisaka, 2018). PEX3 was originally characterized as peroxisomal membrane protein, which cooperates with the cytosolic protein PEX19 in targeting of peroxisomal membrane proteins to pre-existent peroxisomes and in facilitating their membrane insertion (Erdmann et al., 1989; Hettema et al., 2000; Schmidt et al., 2012). As it turned out, however, PEX3 is also present in discrete subdomains of ER membranes and is involved in targeting of certain precursor proteins to ER membranes and most likely in their membrane insertion (Schrul and Kopito, 2016; Yamamoto and Sakisaka, 2018). These precursor proteins include membrane proteins, which either remain in the ER (the two-hairpin or reticulon-domain containing proteins ARL6IP1, RTN3A, RTN4C) or are pinched off in lipid droplets (such as the hairpin protein UBXD8) (Schrul and Kopito, 2016; Yamamoto and Sakisaka, 2018). These observations raised the question if this pathway, too, plays a more global role in protein targeting to the ER (Schrul and Schliebs, 2018; Jansen and Klei, 2019; Dhimann et al., 2020; Goodman, 2020).

Therefore, we addressed the client spectrum of PEX3 in ER protein targeting in human cells and asked if the PEX19/PEX3 pathway to the ER can also target precursor polypeptides to the Sec61 complex (Zimmermann et al., 2021). Here, the approach involved PEX3-depleted HeLa cells and chronically PEX3-deficient Zellweger patient fibroblasts (Schmidt et al., 2012). The negatively affected proteins found in the PEX3 knock-down or knock-out cells included seven peroxisomal membrane proteins and two hairpin proteins of the ER (ATL1, RTN3), thus confirming the two previously identified classes of PEX19/PEX3 clients for ER targeting in human cells (Figure 6; Supplementary Table S1). In addition, 18 membrane proteins (including TA proteins) and 28 proteins with SP (most notably 14 collagens plus collagen-related proteins as well as five membrane proteins) and belonging to the secretory pathway were negatively affected. The latter findings support the notion that PEX3 indeed represents a fourth pathway for targeting of precursor polypeptides to the Sec61 complex. Furthermore, it may suggest a hitherto unknown spatial or at least physical relationship between ER subdomains that are involved in ER shaping and the budding of peroxisomal precursor vesicles, large cargo vesicles, and lipid droplets. Thus in analogy to KTN1, the key observation may be that PEX3 plays a role in targeting of certain precursor polypeptides to Sec61 complexes in ER subdomains.

### 2.4 Translocation of Precursor Polypeptides Into the Human Endoplasmic Reticulum

#### 2.4.1 Sec61 Complex

The heterotrimeric Sec61 complex provides an entry point for precursor polypeptides with SPs into the ER. In the course of co- and posttranslational membrane translocation, the SPs of precursor polypeptides first approach the Sec61 channel (Gumbart and Schulten, 2007; Lang et al., 2017; Lang et al., 2019; Bhadra and Helms, 2021). Subsequently, they begin to sample the cytosolic funnel of the Sec61 channel. According to molecular dynamics simulations, sampling in the Sec61 channel is affected by various properties of precursors or their mRNAs, i.e., deleterious charges, hydrophobicity, mature protein length, arrest peptides or poly-proline motifs in the precursor polypeptides and translation speed, which is dependent on pause sites, rare codons or hairpins in the mRNAs (Zhang and Miller, 2012). For productive SP insertion into and simultaneous full opening of the Sec61 channel, comparatively high hydrophobicity and, therefore, low apparent ΔG value for the H-region was found to be conducive (Gumbart and Schulten, 2007; Zhang and Miller, 2012; Bhadra and Helms, 2021). H-region hydrophobicity of the SP or TMH is supposed to be recognized by the hydrophobic patch formed by four residues of Sec61α TMHs 2 and 7, which line the lateral gate of the channel (Voorhees et al., 2014; Voorhees and Hegde, 2016).

**FIGURE 7:**
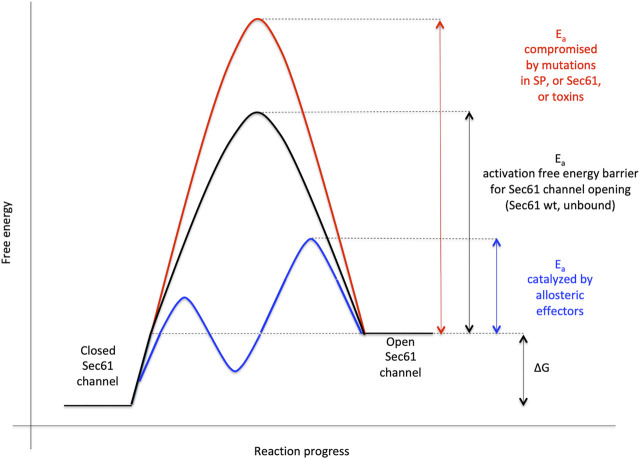
Energetics and kinetics of Sec61 channel gating. In our view, the TRAP- or Sec62/Sec63 +/- BiP-mediated Sec61 channel gating is best visualized in analogy to an enzyme-catalysed reaction. Accordingly, TRAP, Sec62, Sec63 or BiP reduce the energetic barrier for full channel opening, which can apparently be reinforced by Sec61 channel inhibitors, such as cyclic heptadepsipeptides (such as CAM741) or certain eeyarestatins (such as ES24) (Pauwels et al., 2021; Pauwels et al., 2022). At least in the case of ES24, binding of the inhibitor within the channel pore arrests the channel in a partially open state, which may be identical with the primed state and is compatible with Ca^2+^-efflux but not with full channel opening for protein translocation (Gamayun et al., 2019; Bhadra et al., 2021b). TRAP and BiP contribute to full channel opening by direct interaction with ER lumenal loops 5 and 7, respectively, of Sec61α (Figure 1D). *SEC61A1* mutations can increase the free energy barrier for channel opening *per se* (V67G, V85D and Q92R mutation) or indirectly, such as by interfering with BiP binding (Y344H mutation) (for recent reviews see Sicking et al., 2021a; Tirincsi et al., 2022a). Notably, all these effects are precursor specific because the amino-terminal SPs are either efficient or inefficient in driving Sec61 channel opening by themselves. Typical for an enzyme-catalysed reaction, BiP can also support efficient gating of the Sec61 channel to the closed state, i.e. the reverse reaction. The Figure and Figure legend were adapted from Sicking et al. (2021a).

In our opinion, gating of the Sec61 channel can best be described in analogy to an enzyme-catalyzed reaction where the precursor polypeptides with their SPs are the catalysts and the channel is their substrate (Figures 1D, 7) (Haßdenteufel et al., 2018): Channel opening and closing represent two energetically un-favorable reversible reactions and the clients with or without support from auxiliary components or allosteric effectors (TRAP, Sec62/Sec63, see below) are the co-catalysts, which lower the activation energy for the required conformational transitions by binding to the Sec61 complex (Lang et al., 2017; Lang et al., 2019). Interestingly, there are SP mutations of certain precursor polypeptides, such as preproinsulin, preprorenin, as well as *SEC61A1* mutations that can cause the same hereditary diseases, such as Diabetes mellitus and ADTKD (Lloyd et al., 2010; Guo et al., 2014; Bolar et al., 2016; Devuyst et al., 2019; Sicking et al., 2022). Again, this can best be described by an energy diagram for Sec61 channel gating (Figure 7). Accordingly, substitutions of crucial amino acids in either SPs or the pore-forming α-subunit of the Sec61 channel may increase the activation energy for Sec61 channel opening and, therefore, slow down ER import of the particular precursor polypeptide or a whole group of precursor polypeptides, which is particularly dependent on a certain amino acid residue in the Sec61 channel. Notably, *SEC61A1* mutations that cause ADTKD are discussed in the context of additional Sec61-channelopathies in more detail below in the Discussion and were recently reviewed by Sicking et al. (2021a).

**FIGURE 8:**
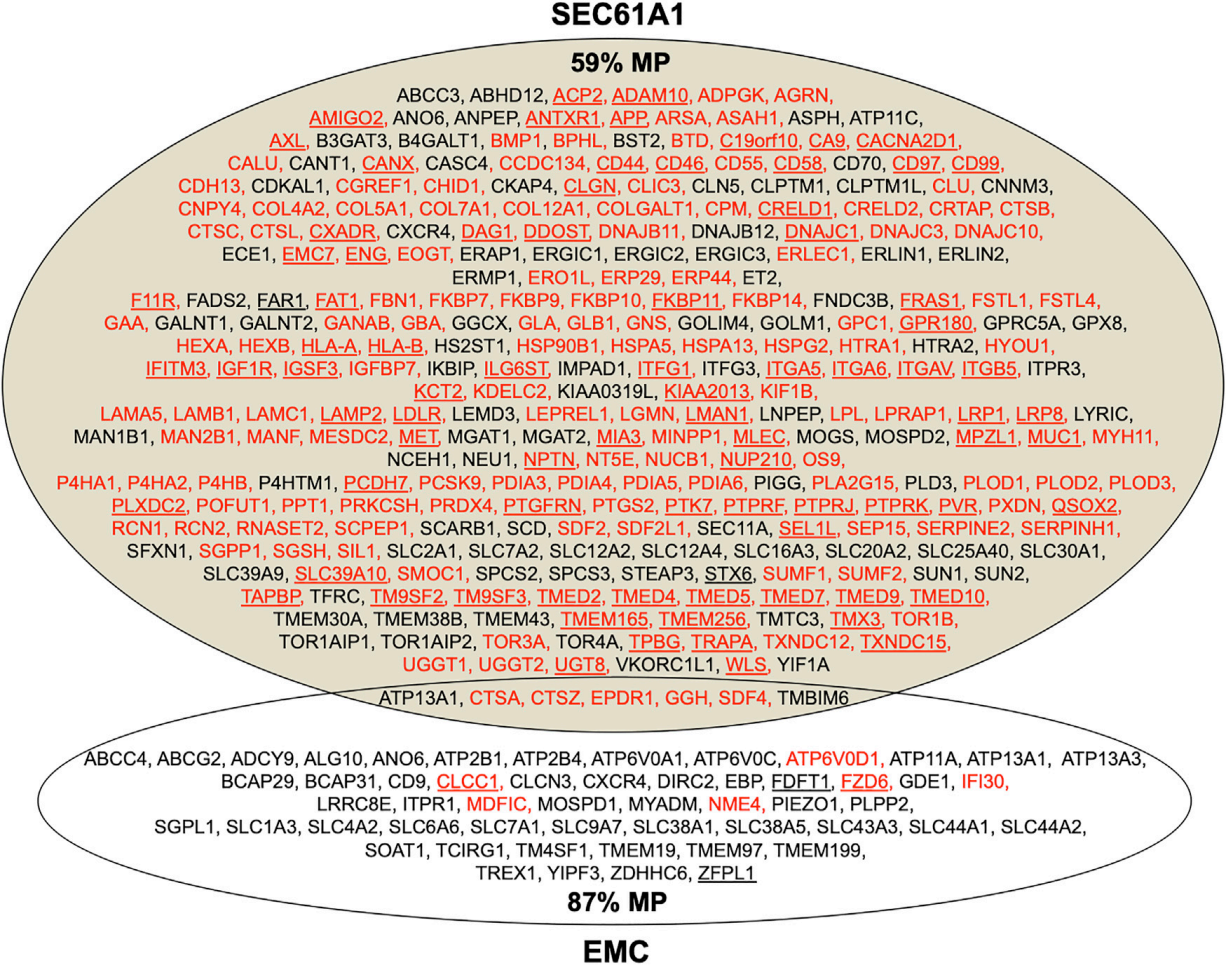
Venn diagram for the Sec61 complex and ER membrane complex (EMC) in the human ER membrane and their clients. The diagram summarizes precursors with SP (in red) or TMH (in black), which are destined to the secretory pathway and were negatively affected by the indicated depletion; membrane protein precursors with SP are underlined, as are TMH proteins with TA. All these precursor polypeptides are defined as potential clients or substrates of the respective component or complex. The section for Sec61 summarizes the negatively affected proteins after the Sec61 depletion in HeLa cells (Nguyen et al., 2018); the section for EMC summarizes the negatively affected proteins after the EMC knock-out in HeLa cells (Shurtleff et al., 2018; Tian et al., 2019). The percentage of membrane proteins (MP) of the secretory pathway among the respective clients is indicated (%).

As mentioned above, the depletion of Sec61α originally served as a proof-of-principle for the proteomic approach (Figures 4, 8; Supplementary Table S1). Among the negatively affected proteins that included all three subunits of the Sec61 complex, GO terms assigned 61% to organelles of the pathways of endocytosis and exocytosis, thus representing a firm enrichment compared to the value for the total quantified proteome (26%) (Nguyen et al., 2018). Furthermore, significant enrichment of precursor proteins with SP (6.8-fold), N-glycosylated proteins (5.6-fold), and membrane proteins (3.0-fold) was detected for the negatively affected proteins (Supplementary Table S1). This suggests that the precursors of these negatively affected proteins, 198 with SP (including 80 membrane protein precursors) and 90 with TMH, represent clients of the Sec61 channel and, therefore, were degraded by the cytosolic proteasome upon Sec61 depletion (Figure 8). As also expected, the positively affected proteins included potential compensatory components, including the two subunits of the SRP receptor (Nguyen et al., 2018). When we analyzed the physicochemical properties of the SPs of the Sec61 clients, precursors with less-hydrophobic SPs were more strongly affected by Sec61 absence, i.e., over-represented in the negatively affected polypeptides, suggesting that precursor polypeptides with a higher SP hydrophobicity are more efficient in Sec61 channel opening than those with lower hydrophobicity. Comparison of Sec61 clients with those of the membrane protein insertase EMC confirmed the preference of the latter for membrane protein precursors (Figure 8) (Shurtleff et al., 2018; Tian et al., 2019). However, the EMC data would also be consistent with the idea that EMC may also be able to facilitate Sec61 channel opening for precursors of soluble proteins with weak SPs, i.e., in analogy to TRAP and Sec62/Sec63 (see below).

#### 2.4.2 Sec62/Sec63 Plus BiP

While the Sec61-complex mediates import of most precursor polypeptides into the ER, the Sec61-associated Sec62/Sec63 heterodimer supports ER protein import in a client-specific manner. Direct interaction between the Sec61 complex and Sec63 was demonstrated by co-immunoprecipitation as well as in living human cells (Tyedmers et al., 2000; Sicking et al., 2021b). Recently, four studies addressed the architecture of the posttranslationally acting translocon complex in yeast by cryo-electron microscopy (cryo-EM) (Itskanov and Park, 2019; Wu et al., 2019; Weng et al., 2021; Itskanov et al., 2021). This particular translocon represents a heptameric protein ensemble, termed the SEC complex (Deshaies et al., 1991). In the SEC complex the trimeric Sec61 complex is assembled with the tetrameric Sec62p/Sec63p complex. The latter comprises two essential, evolutionarily conserved subunits, the membrane proteins Sec62p and Sec63p, and two non-essential subunits, Sec71p and Sec72p. The data provided insights into the mechanism how the SEC complex allows gating of the Sec61 complex and supports ER protein import. Most informative were the observed interactions between Sec63p and the Sec61 complex, which include contacts in the cytosolic, membrane and lumenal domains. Strikingly, the cytosolic Brl domain of Sec63p contacts loops 6 and 8 of Sec61α, thereby blocking the ribosome binding site. Interestingly, as was structurally predicted for the interaction of the TRAPα/β subunits with the Sec61 complex (Pfeffer et al., 2017) and supported by Alphafold 2 (Jumper et al., 2021), the Brl domain of Sec63p represents a canonical beta-sandwich fold to allow an antigen-antibody-like binding to loop 6 of Sec61α. In the membrane, Sec63p (specifically TMH 3 of Sec63) contacts all three subunits of the Sec61 complex in the hinge region opposite of the lateral gate, including TMHs 5 and 1 of Sec61α as well the TMHs of Sec61β and Sec61γ (Figure 1D). Additionally, the short lumenal amino-terminus of Sec63p intercalates on the lumenal side of the channel between the hinge loop 5 of Sec61α and Sec61γ (Itskanov and Park, 2019; Wu et al., 2019; Itskanov et al., 2021; Weng et al., 2021). Apparently, binding of Sec62p/Sec63p to the Sec61 channel causes wide opening of the lateral gate (Van den Berg et al., 2004; Voorhees et al., 2014; Voorhees and Hegde, 2016). The functional implications for the SEC translocon as a consequence of gating by the Sec62p/Sec63p are that SP of many substrates are less hydrophobic and, therefore, have a lower chance to enter the lateral gate and trigger complete opening of the channel. Thus, in the SEC complex binding of the Sec62p/Sec63p induces a fully opened channel that readily accommodates even “weak” or inefficiently gating SPs (Ng et al., 1996; Trueman et al., 2011). Consistent with the concept of the Sec62p/Sec63p inducing wide opening of the lateral gate, yeast Sec62p was found to be able to aid in membrane topology of moderately hydrophobic signal anchor proteins, in particular single-spanning type II membrane proteins, which perform the energetically unfavorable 180° flip turn for correcting their initial type I orientation (Reithinger et al., 2013; Jung et al., 2014; Jung and Kim, 2021).

Similar to yeast, analyses of protein transport in mammalian cells showed a client-specific role of Sec62 in ER protein import. According to *in vitro* experiments with model proteins from insects (such as preprocecropin A) and humans (such as preproapelin and prestatherin), the ER import of presecretory proteins with a content of less than 100 amino acid residues (termed small precursor proteins) into the mammalian endoplasmic reticulum (ER) can occur posttranslationally (Schlenstedt et al., 1990; Shao and Hegde, 2011; Lakkaraju et al., 2012; Johnson et al., 2013; Haßdenteufel et al., 2018) and involves various targeting mechanisms (Haßdenteufel et al., 2018) as well as the ER-membrane proteins Sec62 and Sec63 (Lakkaraju et al., 2012; Lang et al., 2012; Johnson et al., 2013; Haßdenteufel et al., 2017, 2018, and 2019). In case of preprocecropin A, posttranslational ER import has been observed in intact human cells (Shao and Hegde, 2011) and Sec62-dependence of small human presecretory proteins was observed in intact human cells (Lakkaraju et al., 2012).

In contrast to yeast, however, the mammalian Sec62 protein apparently experienced a gain of function, i.e., it can interact with ribosomes near the ribosomal tunnel exit and can support cotranslational transport of certain clients, such as the precursors of ERj3- and prion-protein with 358 and 253 amino acid residues, repectively (Müller et al., 2010; Ziska et al., 2019; Schorr et al., 2020). Therefore, crosslinking experiments with stalled precursor polypeptides in transit through the mammalian translocon observed the dynamic recruitment of allosteric Sec61 channel effectors, such Sec62 (Conti et al., 2015). In contrast, the model precursor bovine preprolactin triggered Sec61 recruitment of accessory factors such as the allosteric effector TRAP and the auxiliary translocating chain-associating membrane (TRAM) protein. However, when ERj3- or prion-protein were used as model transport substrates the Sec62/Sec63 instead of TRAP and TRAM were recruited to the channel in order to allow translocation of substrates having a weak or inefficiently gating SP (Conti et al., 2015). In other studies, another dynamic transition of the translocon was observed for Sec62 and the SRP receptor. To allow cotranslational targeting the SR can displace Sec62 from the Sec61 complex, thereby switching the Sec61 channel from Sec62- to SRP-dependent translocation (Jadhav et al., 2015). According to the above mentioned crosslinking approach, however, SR and Sec62 can also act sequentially, namely after SRP-dependent targeting of precursors of ERj3- and prion-protein, Sec62 can displace SR from the Sec61 channel and -together with Sec63- support channel opening. Furthermore, Sec63 has to “take over” loops 6 and 8 of Sec61α from the ribosome. The cryo-EM structures of the yeast SEC complex may support the idea of a dynamic transition and flexibility of Sec62/Sec63. Both Sec62p and the ER lumenal J-domain of Sec63p could not be sufficiently resolved in the single particle analysis and this might have been due to their structural flexibility and dynamic assembly into the SEC translocon.

Notably, for the prion protein precursor, Sec62/Sec63-dependent ER-import has also been demonstrated in a genetic screen in human cells (Davis et al., 2015). Subsequent *in vitro* import assays, using a full-length prion protein precursor, demonstrated SRP-dependence and the fact that Sec63-dependence is not only due to the SP but also due to a polybasic motif, which is downstream of the SP in the mature region (Ziska et al., 2019) and was missing from the artificial prion protein construct in previous work (Rane et al., 2009; Davis et al., 2015). Furthermore, these *in vitro* import assays demonstrated that Sec63-dependence of the small preproapelin and the precursors of ERj3 and prion protein is related to gating of the Sec61 channel to the open state and coincides with BiP´s involvement, which was linked to the combination of a weak SP plus, in case of preproapelin (_37_RRK) and the prion protein precursor (_1_KKRPK), a positively charged cluster in the mature region (Haßdenteufel et al., 2018; Ziska et al., 2019). Notably, loss of Sec63 protein function in the liver of a subset of human patients with polycystic liver disease was also interpreted in light of a client specific function of Sec63 in ER protein import (Fedeles et al., 2011; Lang et al., 2012). Interestingly, Sec63-dependence of ERj3 import into the ER was indirectly confirmed in murine *SEC63* null cells, which were generated as an animal model for the human disease (Lang et al., 2012). These *SEC63* ^−/−^ cells lacked ERj3 while the levels of various other ER proteins were unchanged compared to murine *SEC63* ^+/+^ cells (Fedeles et al., 2011; Schorr et al., 2020).

**FIGURE 9:**
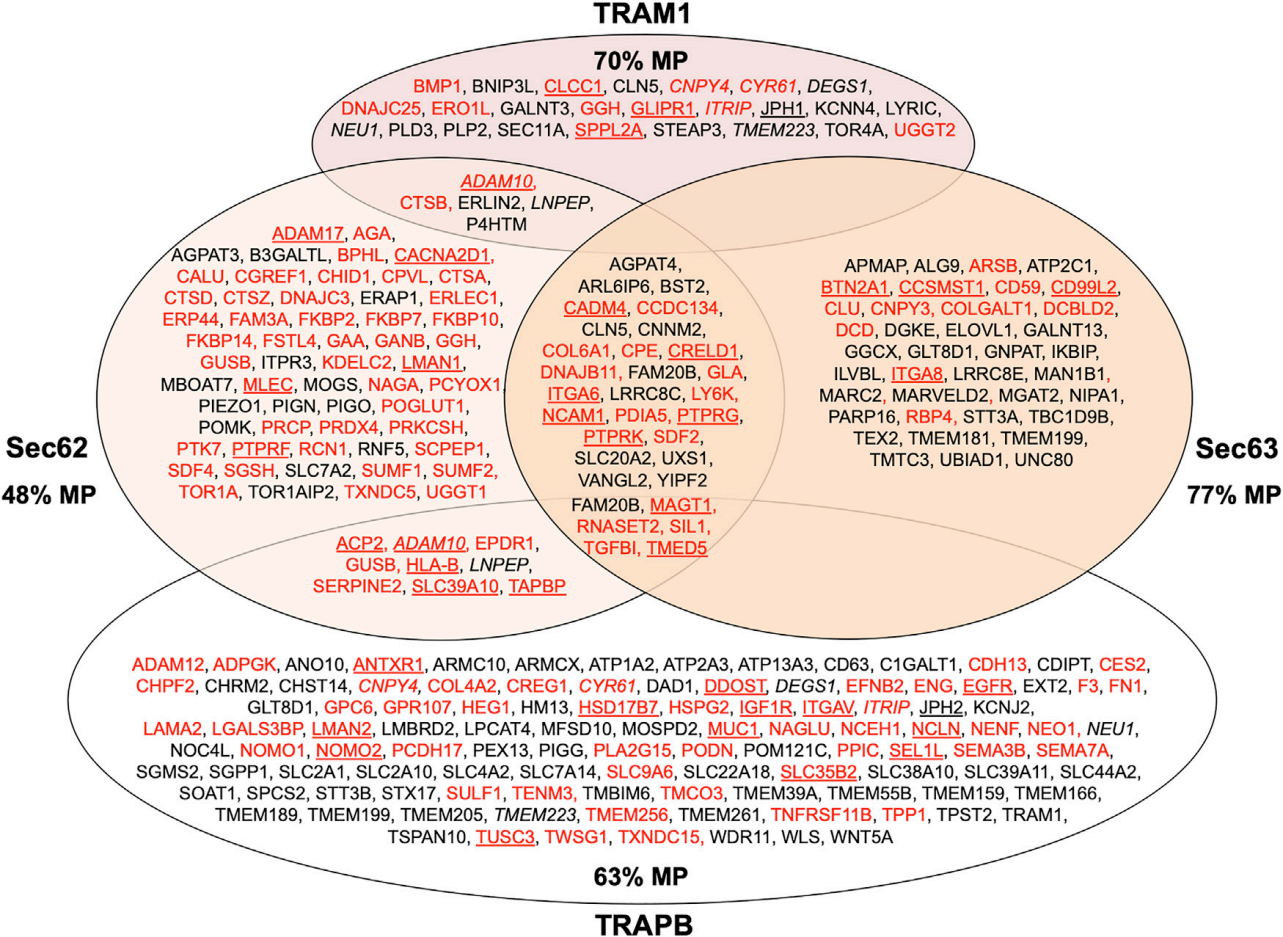
Venn diagram for human ER protein translocation components and their clients. The diagram summarizes precursors with SP (in red) or TMH (in black), which are destined to the secretory pathway and were negatively affected by the indicated depletion; membrane protein precursors with SP are underlined, as are TMH proteins with TA. All these precursor polypeptides are defined as potential clients or substrates of the respective component or complex. The section for Sec62 and Sec63 summarizes the negatively affected proteins after the respective depletions in HeLa cells and the knock-outs in HEK293 cells, respectively (Schorr et al., 2020); the section for TRAM1 summarizes the negatively affected proteins after the TRAM1 depletion in HeLa cells (Klein et al., 2020); the section for TRAP summarizes the negatively affected proteins after the TRAPB depletion in HeLa cells and the TRAPD or TRAPG knock-outs in CDG patient fibroblasts (Nguyen et al., 2018). The percentage of membrane proteins (MP) of the secretory pathway among the respective clients is indicated (%). ADAM10, CNPY4, CYR61, ITRIP, LNPEP and TMEM223 are in italics to highlight that they were negatively affected by TRAM1 as well as TRAPB depletion.

With our proteomic approach, we determined the rules for engagement of Sec62/Sec63 in ER-import in intact human cells (Schorr et al., 2020). Applying the statistical analysis, we found that Sec62 depletion significantly affected the steady-state levels of 351 proteins: 155 negatively and 196 positively (Figure 9; Supplementary Table S2). The identified precursors included 18 proteins with cleavable SP and six proteins with TMH. The proteins positively affected by transient Sec62 depletion included both SRP- receptor subunits (SRPRA, SRPRB) and the TRAP ß-subunit (coded by the SSR2 gene). We assume that these short-term compensatory mechanisms may have contributed to the comparatively low number of negatively affected proteins. The Sec63-depletion significantly affected the steady-state levels of 34 proteins: 21 negatively and 13 positively (Figure 9; Supplementary Table S2). The identified precursors included four proteins with cleavable SP and six proteins with TMH and only one of the proteins with SP had also been negatively affected by Sec62-depletion (TGFBI). Upon closer inspection of the potential overlap between Sec62 and Sec63 depletion in HeLa cells, four additional precursor polypeptides with SP were negatively affected by Sec63-depletion in HeLa cells, which, however, did not meet the significance threshold (ERj3 –coded by the DNAJB11 gene-, MAGT1, PDIA5, SDF2) (Schorr et al., 2020).

To identify additional substrates, we performed similar analyses after Sec62 or Sec63 knock-out, employing CRISPR/Cas9 treated HEK293 cells compared to HEK293 control cells (Fumagalli et al., 2017; Schorr et al., 2020). Here, we found that Sec62 deficiency significantly affected the steady-state levels of 329 proteins: 208 negatively and 121 positively (Supplementary Table S2). Of the negatively affected proteins, GO terms assigned ∼48% to organelles of the endocytic and exocytic pathways. We also detected significant enrichment of proteins with SP (5.5-fold), N-glycosylated proteins (4.5-fold), and membrane proteins (1.8-fold). The identified precursors included 74 proteins with cleavable SP (including 19 membrane proteins) and 29 proteins with TMH. As expected (Conti et al., 2015), ERj3 was negatively affected. After Sec63 knock-out in HEK293 cells, we found that Sec63-deficiency significantly affected the steady-state levels of 302 proteins: 199 negatively and 103 positively (Supplementary Table S2). GO terms assigned ∼37% of the negatively affected proteins to organelles of the endocytic and exocytic pathways. We also detected significant enrichment of proteins with SP (1.9-fold), N-glycosylated proteins (2.4-fold), and membrane proteins (1.8-fold). The identified precursors included 24 proteins with cleavable SP (including ten membrane proteins) and 38 proteins with TMH (Figure 9). Here, 22 precursor polypeptides were negatively affected by Sec62 as well as Sec63 deficiency, 11 each with SP (including six membrane proteins) and with TMH, all belonging to the secretory pathway (Figure 9). Upon closer inspection of the potential overlap between Sec62 and Sec63 in HEK293 cells, six additional precursor polypeptides with SP were negatively affected by Sec63-deficiency, which did not meet the significance threshold (MAGT1, PDIA5, RNASET2, SDF2, SIL1, TMED5) (Schorr et al., 2020). Interestingly, the analysis also identified 54 precursors with SP (including 11 membrane proteins) plus 17 precursors with TMH, which showed a requirement for Sec62 but not for Sec63, as well as 12 precursors with SP (including four membrane proteins) plus 26 precursors with TMH, which showed a requirement for Sec63 but not for Sec62, consistent with the previous observation *in vitro* that the two proteins can also support ER protein import independently of each other (Haßdenteufel et al., 2018). Notably, the overlap was probably underestimated since the analysis was done for mixed guide RNA clones in the case of Sec63, i.e., the cell pool did not represent a SEC63 knock-out (Schorr et al., 2020).

**FIGURE 10:**
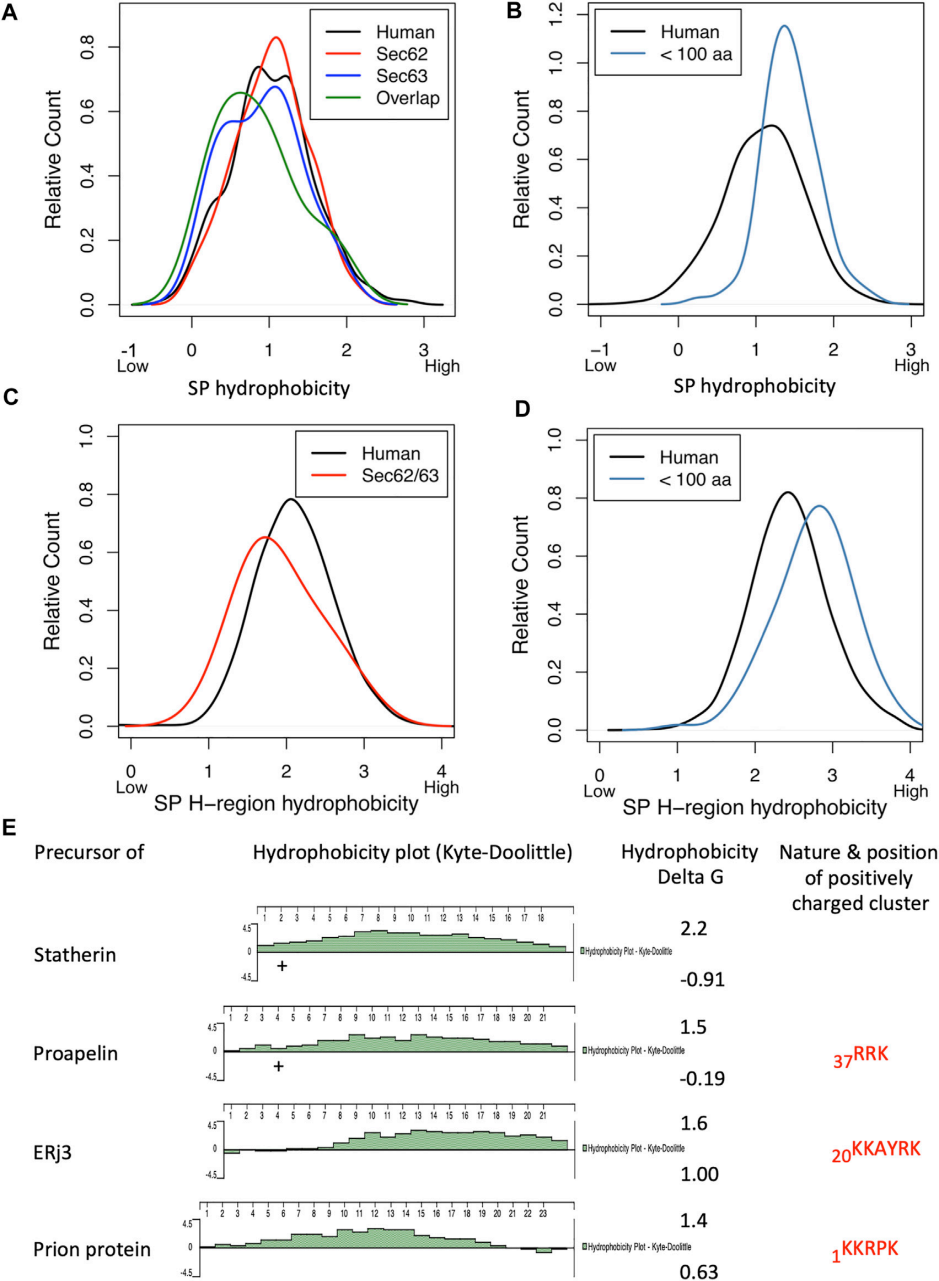
Physicochemical properties of Sec62/Sec63 clients. **(A–C)** We used custom scripts to compute the hydrophobicity of SPs **(A,B)** and SP H-regions **(C,D)**, respectively. Hydrophobicity score was calculated as the averaged hydrophobicity of its amino acids according to the Kyte-Doolittle propensity scale. For the calculation of H-region hydrophobicity, each SP was subjected to segmentation using the prediction tool Phobius (https://phobius.sbc.su.se) and the H-region to the calculation according to Kyte-Doolittle. **(E)** Relevant properties of SPs (hydrophobicity and positively charged amino acid residues, respectively) and mature regions of four clients. Hydrophobicity scores were calculated according to the Kyte-Doolittle propensity scale and are displayed using the DNAstar software package. Apparent delta G values were determined with the ΔG_app_ predictor for TM helix insertion (http://dgpred.cbr.su.se). Clusters of positive charges in the respective mature region that were experimentally shown to contribute to Sec62/Sec63 dependence are indicated.

Along with confirming ERj3 as a client, 30 novel Sec62/Sec63-clients were identified under these in vivo-like conditions, 18 with SP (including eight membrane proteins) and 12 with TMH (Figure 9; Supplementary Table S2). These previously unknown substrates have in common less hydrophobic SPs with longer but less hydrophobic H-regions and lower C-region polarity (Figures 10A,C). Further analyses with four substrates, ERj3 in particular, revealed the combination of a weak SP and a translocation-disruptive positively charged cluster of amino acid residues within the mature part (_20_KKAYRK) as decisive for the Sec62-/Sec63-requirement (Figure 10E) (Schorr et al., 2020). This is reminscent of preproapelin and prion protein import (Haßdenteufel et al., 2018; Ziska et al., 2019) and in all three cases these features were found to be responsible for an additional BiP-requirement and for sensitivity towards the Sec61 channel inhibitor CAM741. Thus, human Sec62/Sec63 may support Sec61 channel opening for precursor polypeptides with weak SPs by direct interaction with Sec61α and/or via recruitment of BiP and its interaction with the ER-lumenal loop 7 of Sec61α, which we supposed to lower the activation energy for channel opening (Figure 7).

#### 2.4.3 ERj1 Plus BiP

ERj1 belongs to the class of ribosome-associated membrane proteins (RAMPs) (Dudek et al., 2002; Blau et al., 2005; Dudek et al., 2005; Benedix et al., 2010). However, its ribosome association, appears to be more dynamic as compared to the classical RAMPs, i.e., Sec61, TRAM and TRAP (Görlich et al., 1992b; Görlich and Rapoport, 1993). This was microscopically confirmed by fluorescence microscopy using fluorescently labeled antibodies against ERj1 in permeabilized MDCK cells (Snapp et al., 2004; Benedix et al., 2010). According to cryo-EM, the cytosolic domain of ERj1 binds at the ribosomal tunnel exit and involves expansion segment 27 (ES27) of the 28S rRNA (Blau et al., 2005). ERj1 was proposed to play a role in ER protein import as a possible functional homolog for Sec62/Sec63, combining the cytosolic ribosome binding activity of Sec62 with the ER lumenal Hsp40-type co-chaperone activity of Sec63 in one polypeptide (Dierks et al., 1996; Skowronek et al., 1999; Mayer et al., 2000; Tyedmers et al., 2000; Dudek et al., 2005; Tyedmers et al., 2005; Müller et al., 2010; Lang et al., 2012; Schäuble et al., 2012). Notably, human Sec62 was microscopically confirmed as RAMP, too, by fluorescence microscopy using fluorescently labeled antibodies against Sec62 in permeabilized MDCK cells (Snapp et al., 2004; Müller et al., 2010). Interestingly, the cytosolic domain of ERj1 is able to allosterically inhibit translation at the stage of initiation when its ER lumenal J-domain is not associated with BiP but allows translation when BiP is bound (Benedix et al., 2010). Thus, ERj1 would be perfectly able to allow initiation of protein synthesis of precursor polypeptides on ER bound ribosomes when BiP is available on the ER lumenal side of the membrane.

Employing the statistical analysis, we found that transient and partial ERj1 depletion significantly affected the steady-state levels of 172 proteins: 92 negatively and 80 positively (Figure 5; Supplementary Table S2) (Bhadra et al., 2021a). Of the negatively affected proteins, GO terms assigned almost 30% to organelles of the pathways of endocytosis and exocytosis. The identified precursors included seven proteins with cleavable SP, among them two membrane proteins, and eight membrane proteins with TMH and were discussed above in the context of KTN1-dependent mRNA targeting to the ER.

#### 2.4.4 TRAP

Originally, TRAP was characterized as signal-sequence receptor (SSR) complex (Wiedmann et al., 1987). Furthermore, it had been crosslinked to nascent polypeptides at late translocation stages (Conti et al., 2015) and had been demonstrated to associate with Sec61 (Menetret et al., 2008; Dejgaard et al., 2010; Pfeffer et al., 2017). As mentioned in the Introduction, the ribosome-associated Sec61-complex and the TRAP form a stable stoichiometric super-complex called a translocon (Menetret et al., 2008; Bano-Polo et al., 2017; Pfeffer et al., 2017). *In vitro* transport studies showed that the TRAP stimulates protein translocation depending on the efficiency of the SP in transport initiation (Fons et al., 2003); Sec61 gating efficiency and TRAP dependence were inversely correlated. Recent studies in intact cells suggest that TRAP may also affect TMH topology (Sommer et al., 2013), reminiscent of Sec62/Sec63 in yeast (Reithinger et al., 2013; Jung et al., 2014).

To identify TRAP dependent precursors, we combined siRNA-mediated TRAP depletion in HeLa cells, label-free quantitative proteomics, differential protein abundance analysis, and statistical analysis. We found that TRAPβ depletion significantly affected the steady-state levels of 257 proteins: 180 negatively and 77 positively. Of the negatively affected proteins, GO terms assigned ∼40% to organelles of the endocytotic and exocytotic pathways and included all four subunits of TRAP. We also detected significant enrichment of proteins with SP (3.3-fold), N-glycosylated proteins (2.7-fold), and membrane proteins (2.1-fold). The identified precursors included 38 proteins with cleavable SP and 22 proteins with TMH, and represented N-glycosylated proteins and non-glycosylated proteins (Figure 9; Supplementary Table S2). For TRAP deficient fibroblasts from patients with a Congenital disorder of glycosylation the steady-state levels of 318 proteins were altered: 279 negatively and 39 positively (Supplementary Table S2). Of the negatively affected proteins, GO terms assigned 36% to organelles of the pathways of endocytosis and exocytosis. The identified precursors included 34 proteins with cleavable SP and 41 proteins with TMH. Taken together, TRAP knock-down and knock-out identified 59 membrane proteins with TMH and 66 proteins with SP (including 20 membrane proteins), all belonging to the secretory pathway, as TRAP clients (Figure 9). Interestingly, six of the TRAP clients were found among the Sec62/Sec63 clients and nine among the Sec62 substrates, consistent with the view that these two allosteric Sec61 channel effectors have overlapping but non-identical functions.

**FIGURE 11:**
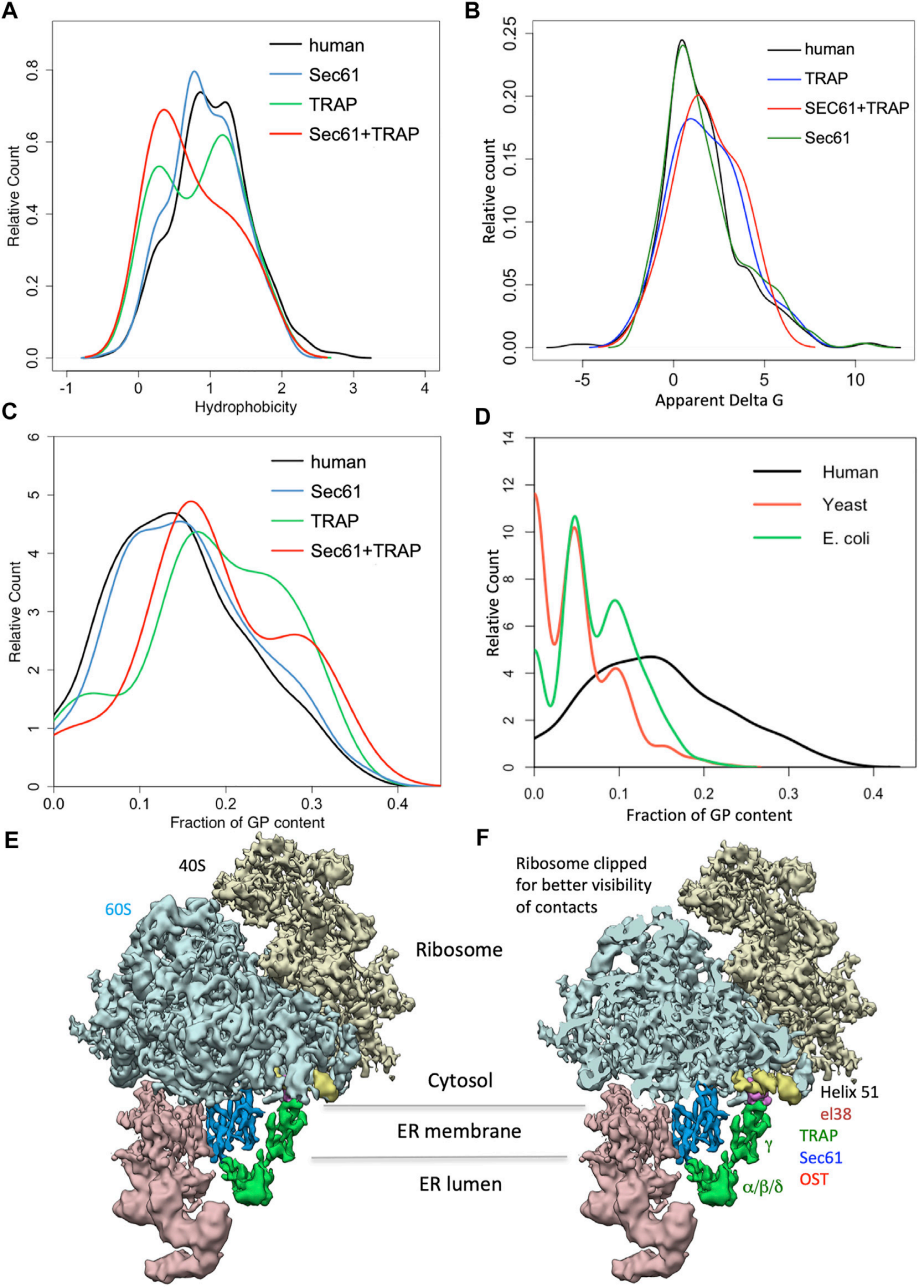
Physicochemical properties of SPs of TRAP clients. **(A–C)** We used custom scripts to compute the hydrophobicity score **(A)**, apparent delta G **(B)**, and glycine/proline (GP) content **(C)** of SP sequences. Hydrophobicity score was calculated as the averaged hydrophobicity of its amino acids according to the well-known Kyte-Doolittle propensity scale. Apparent delta G values were determined with the ΔG_app_ predictor for TM helix insertion (http://dgpred.cbr.su.se). GP content was calculated as the total fraction of glycine and proline in the respective sequence. **(D)** We also used custom scripts to extract protein annotations for all human, *E. coli* and *S. cerevisiae* SPs from UniProtKB entries and to calculate their GP content. **(E,F)** Cartoon of unclipped **(E)** and clipped **(F)** 80S ribosome together with Sec61-complex and TRAP, and OST. Notably, without clipping eL38 and helix 51 is partially hidden.

The SP analysis of TRAP-substrates demonstrated an above-average glycine-plus-proline content (GP content) and below-average hydrophobicity as the key features (Figures 11A–C). Thus, the Sec61-associated TRAP supports protein translocation in a substrate-specific manner. We suggest that high GP content and low hydrophobicity extend the dwell time of SP at the cytosolic funnel of the Sec61 channel, and that TRAP can compensate this potential problem by stabilizing SP on the cytosolic surface and by aiding in Sec61 channel gating at the lumenal side. This raises the question of how TRAP relays the presence of an SP-bearing RNC to the Sec61 channel. In an attempt to interpret our findings at the structural level, i.e., in the context of the TRAP architecture, in which individual TRAP subunits were assigned positions within the overall density of human TRAP in native ER membranes by cryo-electron tomography (CET) (Figures 11E,F) (Pfeffer et al., 2017), the ER-lumenal domains of the TRAPαβ-subcomplex contact loop 5 in the hinge region between the amino- and carboxy-terminal halves of Sec61α and, thereby, mediate Sec61 channel opening by lowering the activation energy, required for channel opening (Figure 7). TRAPγ occupies a central position in human TRAP, contacting eL38 and short rRNA expansion segment (ES) on the ribosome, thus coordinating the other TRAP subunits with the ribosome and the additional translocon components, i.e. the Sec61-complex (contacted by TRAPαβ) and OST (contacted by TRAPδ). Previously, the ribosomal components uL24 and H59, both in vicinity to eL38 and TRAPγ, were observed to coordinate SP for SRP binding in the bacterial system (Jomaa et al., 2016). Assuming a similar SP position in the human system, the amino-terminal SP tip may consequently be close enough to interact with eL38 and the cytosolic domain of TRAPγ during the “hand-over” of the SP from SRP to Sec61 (Figure 11E). According to this hypothetical scenario, TRAP may support the insertion of SP into the Sec61 channel in the productive hairpin (rather than head-first) configuration.

#### 2.4.5 TRAM1

TRAM (since the discovery of TRAM2 (Stefanonvic et al., 2004) termed TRAM1) represents an ER membrane protein with eight TMHs. It belongs to a protein family, characterized by the TLC (short for: TRAM/LAG1/CLN8) homology domain, which is supposed to bind ceramide or related sphingolipids (Klein et al., 2020). Similarly to TRAP, it was discovered by crosslinking of nascent presecretory proteins in the context of RNCs, but in contrast to TRAP early in their translocation into the ER (Görlich et al., 1992b; Görlich and Rapoport, 1993). Subsequently, it was described to interact with nascent membrane proteins in the course of their initial integration into the Sec61 channel (High et al., 1993; Mothes et al., 1994; Do et al., 1996; Voigt et al., 1996; Hegde et al., 1998; McCormick et al., 2003; Sadlish et al., 2005; Sauri et al., 2007). Actually, TRAM1 was one of the first proteins found to provide substrate-specific support for ER protein import (Görlich and Rapoport, 1993). Furthermore, it was observed that precursor proteins with short charged amino-terminal domains in their SPs require TRAM1 for efficient insertion into the lateral gate and that TRAM1 can regulate cytosolic extrusion of nascent chain domains into the gap between ribosome and translocon (Voigt et al., 1996; Hegde et al., 1998). In addition, it was concluded that precursors with shorter than average N-regions and shorter H-regions in their SP require the help of TRAM1 for efficient insertion into the lateral gate.

By applying our unbiased proteomic approach, we identified 30 potential TRAM1 substrates that included 13 precursors with SP (including four membrane proteins) and 17 with TMH (Figure 9; Supplementary Table S2) (Klein et al., 2020). Comparing these precursors to those found for Sec61 and TRAP in similar experiments, did not point to a preference of TRAM1 for any particular type of precursor polypeptides. Furthermore, analysis of the physicochemical properties of SP and TMH of the TRAM1 substrates did not point to a specific feature, except for precursors with short N-regions in their SP. Notably, 27% of the TRAM1 substrates were also negatively affected by TRAP depletion (Figure 9). This is consistent with the co-localization of TRAM1 with Sec61 and TRAP (Görlich and Rapoport, 1993; Dejgaard et al., 2010). Considering the strong overlap in substrates of these two transport components and TRAM1’s apparent lack of precursor preference may indicate that TRAM1 does not act as a receptor for SPs and TMHs. It may rather play a supportive role in ER protein import, such as making the phospholipid bilayer conducive for accepting SP and TMH in the vicinity of the lateral gate of the Sec61 channel. This interpretation is consistent with the above-mentioned prediction that TRAM1 may be able to bind sphingolipids (Klein et al., 2020).

## 3 Discussion

In human cells, approximately 30% of all polypeptides enter the secretory pathway at the level of the ER. This process involves SPs or equivalent TMHs at the level of the precursor polypeptides and a multitude of cytosolic and ER proteins, which guarantee the initial ER targeting as well as the subsequent membrane integration or translocation (Figure 2). Cytosolic SRP and SR in the ER membrane mediate cotranslational targeting of most nascent precursor polypeptide chains to the polypeptide-conducting Sec61 complex in the ER membrane. Alternatively, nascent and fully-synthesized precursor polypeptides are targeted to the ER membrane by either the PEX3/19-, SND-, or TRC-pathway and mRNAs are targeted to the ER membrane by nucleic acid-based pathways. According to the classical *in vitro* studies for ER protein import, these targeting pathways may have overlapping functions, which raised the question how relevant this is under cellular conditions and which features of SPs and/or entire precursor polypeptides determine preference for a certain pathway under these conditions. Irrespective of their targeting pathway(s), most precursor polypeptides are integrated into or translocated across the ER membrane via the Sec61 channel. For some precursors Sec61 interaction partners have to support the gating of the channel, again raising the question why and when this is the case, i.e., what the client specificities of these auxiliary components are, i.e., Sec62/Sec63, TRAM1 protein, TRAP. In the course of the last 5 years, we combined siRNA-mediated depletion or knock-out of single targeting or transport components in human cells with label-free quantitative proteomics and differential protein abundance analysis to characterize client specificities of these components. Here, we present a summary of the clients, which were identified in the respective differential protein abundance analyses and highlight some of the lessons learned.

In mRNA targeting to the human ER, the putative receptors AEG-1 and RRBP1 show considerable overlap in their clients, which are directed towards the secretory pathway (Figure 5) (Hsu et al., 2018; Bhadra et al., 2021a). The results for KTN1 suggest a possible function of KTN1 (in possible cooperation with ERj1 and BiP) as the hitherto elusive ER membrane-resident mRNA receptor in the so-called TIGER domain, which may form a cytosolic micro-domain that enriches certain membrane protein- as well as cytoskeletal protein-encoding mRNAs with multiple AU-rich elements (AREs, specifically ATTTA motifs) in their 3′ UTRs in the vicinity of the ER (Berkovits and Mayr, 2015; Ma and Mayr, 2018; Bhadra et al., 2021a). Indeed multiple ATTTA motifs were found in the 3′ UTRs of several mRNAs and, therefore, appear to be one but certainly not the only distinguishing feature in this process.

In targeting of precursor polypeptides to the human ER, the results from the classical *in vitro* studies for ER protein import were confirmed, i.e. all four known targeting pathways were found to be able to target SPs and TMHs to the Sec61 complex in the ER membrane. When the respective SPs were analyzed with various analytical tools, no significant distinguishing features were determined. However, for the PEX3/PEX19-dependent pathway, which plays its major roles in targeting peroxisomal membrane proteins and certain hairpin membrane proteins of the ER and lipid droplets to a hitherto ill-defined ER subdomain (Schrul and Kopito, 2016; Yamamoto and Sakisaka, 2018), the analysis suggested that this subdomain may be physically or even spatially related to ER exit sites for large cargo vesicles, which are crucial for collagen secretion (Zimmermann et al., 2021). Therefore, various collagens as well as collagen-modifying enzymes and interacting proteins, most of them with SP, were found to be targeted to this subdomain by unknown features. We proposed that the defects in the biogenesis of certain collagens may contribute to the devastating effects of PEX3 deficiency in Zellweger patients. As expected, there were no TA membrane proteins found among the SRα clients and the SRP/SR-dependent pathway showed the expected preference for precursors with N-terminal SP or more amino-terminal TMH (Tirincsi et al., 2022b). In contrast to both the PEX3/PEX19- and SRP/SR-dependent pathways, TRC- and SND-dependent ER protein targeting showed a preference for multi-spanning membrane proteins as well as for membrane proteins with central or carboxy-terminal TMHs (Tirincsi et al., 2022b). These findings may explain why the latter two pathways can substitute for each other to a certain extent. Furthermore, they are consistent with the observations that there is a considerable overlap in clients between the latter two pathways and hardly any overlap with the other two pathways.

With respect to protein translocation into the human ER, precursors with less-hydrophobic SP were more strongly affected by Sec61 depletion, i.e. over-represented among the negatively affected polypeptides (Nguyen et al., 2018). Thus, precursor polypeptides with a higher-than-average SP hydrophobicity appear to be more efficient in Sec61 channel opening than those with lower hydrophobicity, which may be linked to the characteristics of the hydrophobic patch formed by four residues of Sec61α TMHs 2 and 7 that line the lateral gate of the channel and are crucial for its opening (Voorhees et al., 2014; Voorhees and Hegde, 2016). In addition, SP hydrophobicity was observed to be crucial for the roles of the so-called allosteric effectors of the Sec61 channel, TRAP and Sec62/Sec63 plus BiP, in channel opening, thereby confirming conclusions from *in vitro* experiments and extending them to the cellular level. This may explain why the two auxiliary complexes share some substrates (Figure 9). For SPs having low overall hydrophobicity in combination with high glycine- plus proline-content, i.e. low alpha-helical propensity, full Sec61 channel opening in cotranslational transport was found to be supported by TRAP (Nguyen et al., 2018), a SP feature that had not been previously appreciated. Furthermore, to accommodate SPs with low H-region hydrophobicity, particularly in combination with detrimental features within the mature part, full Sec61 channel opening was observed to be supported by Sec62/Sec63 with or without BiP involvement (Ziska et al., 2019; Schorr et al., 2020). This raises the questions why this is the case and what the possible benefits are. To answer the second question first, we suggested that these features may allow differential regulation of ER protein import under different cellular conditions, for example by the known phosphorylation or Ca^2+^ binding of the respective transport components (Table 1). Sec63 and Sec62 were described to be subject to phosphorylation and Ca^2+^-binding, respectively (Ampofo et al., 2013; Linxweiler et al., 2013). Thus, these modifications are candidates for Sec62/Sec63- and ER protein import-regulation, i.e. the different requirements of different precursors may provide a basis for dual intracellular location of proteins, such as ERj6 –coded by the DNAJC3 gene- (Shaffer et al., 2005; Oyamadori et al., 2006; Rutkowski et al., 2007; Petrova et al., 2008), a Sec62-client in HEK293 cells and in HeLa cells (Schorr et al., 2020). Furthermore, ERj1 was found to be subject to phosphorylation (Götz et al., 2009) and TRAPα was found to be subject to phosphorylation as well as Ca^2+^-binding (Wada et al., 1991) and, therefore, may reciprocally respond to the same cellular conditions as compared to Sec62/Sec63. We are convinced that the detected variations in SP and TMH characteristics are responsible for the known precursor specific defects in various human diseases, termed Sec61-channelopathies (reviewed by Haßdenteufel et al., 2014; Sicking et al., 2021a), which include *SEC61A1*-linked Common variable immunodeficiency (Schubert et al., 2018), Neutropenia (Van Nieuwenhove et al., 2020) and Tubulointerstitial kidney disease (Bolar et al., 2016; Sicking et al., 2022), *SEC61B*- and *SEC63*-linked Polycystic liver disease (Fedeles et al., 2011; Lang et al., 2012; Besse et al., 2017), and *SSR*- as well as *CAML*-linked Congenital disorders of glycosylation (Pfeffer et al., 2017; Nguyen et al., 2018; Wilson et al., 2022) (Table 1).

To address the first question, it is noteworthy that higher than average overall hydrophobicity and higher than average H-region hydrophobicity seem to define “weak” or inefficiently gating SPs in the context of small precursor proteins (Haßdenteufel et al., 2019) (Figures 10B,D), which is in sharp contrast to the SP of precursor polypeptides in cotranslational translocation mentioned above (Figures 10A,C). Therefore, the question is how these contradictory findings can be reconciled. We suggest that both higher and lower than average SP hydrophobicity extends the dwell time of these SPs at the cytosolic funnel of the Sec61 channel, simply because their interactions with the hydrophobic patch are either too strong, i.e., disfavouring reversibility, or not strong enough to trigger spontaneous opening of the lateral gate and accompanying full channel opening, which may best be envisioned in the energy diagram for Sec61 channel gating (Figure 7). Therefore, these features were found to be responsible for the additional BiP-requirement in the case of the precursors of ERj3 (Schorr et al., 2020), prion protein (Ziska et al., 2019), and proapelin (Haßdenteufel et al., 2018), and the sensitivity towards the Sec61 channel inhibitor CAM741. This SP effect appears to be reinforced by clusters of positive charges downstream of the SP in co- and posttranslational translocation (Figure 10E) (Haßdenteufel et al., 2018; Ziska et al., 2019; Schorr et al., 2020). Therefore, allosteric Sec61 channel effectors have to bind to the channel, which supposedly lowers the activation energy for channel opening, in particular when aberrant SP hydrophobicity coincides with low SP helix propensity, as in the case of TRAP (Nguyen et al., 2018), or with deleterious features downstream of the SP in the mature region, as in the case of Sec62/Sec63 (Schorr et al., 2020). According to the available structural data, both accessory complexes, Sec62/Sec63 and TRAP, appear to act on the Sec61 channel on its lumenal side, i.e., in proximity to loop 5, which connects the amino- and carboxy-terminal halves of Sec61α. Thus, interaction of the accessory complexes with loop 5 might support the rigid body movement in the course of Sec61 channel opening. When BiP is involved in channel opening in addition to Sec62/Sec63, it is recruited to the Sec61 complex by Sec63, binds to ER lumenal loop 7 of Sec61α, and contributes to the lowering of the activation energy for channel opening (Schäuble et al., 2012; Haßdenteufel et al., 2018).

Considering the evolutionary conservation of the GP content of SPs encountered in TRAP-containing humans and TRAP-free organisms such as yeast and *E. coli* points to a much higher GP content in the former (Figure 11D). Thus, enabled by TRAP, the human Sec61 channel can manage SPs with a higher content of glycines and prolines, i.e., a lower helix propensity, compared to its homologous ancestors in yeast and bacteria. Such a scenario speaks in favor of a co-evolution of SPs and allosteric effectors of the Sec61 complex eventually allowing for a broader client spectrum and a more complex orchestration of protein transport.
